# MLH1 Region Polymorphisms Show a Significant Association with CpG Island Shore Methylation in a Large Cohort of Healthy Individuals

**DOI:** 10.1371/journal.pone.0051531

**Published:** 2012-12-11

**Authors:** Andrea J. Savio, Mathieu Lemire, Miralem Mrkonjic, Steven Gallinger, Brent W. Zanke, Thomas J. Hudson, Bharati Bapat

**Affiliations:** 1 Department of Laboratory Medicine and Pathobiology, University of Toronto, Toronto, Ontario, Canada; 2 Samuel Lunenfeld Research Institute, Toronto, Ontario, Canada; 3 Ontario Institute for Cancer Research, Toronto, Ontario, Canada; 4 Ontario Familial Colorectal Cancer Registry, Cancer Care Ontario, Toronto, Ontario, Canada; 5 Department of Surgery, University of Toronto, Toronto, Ontario, Canada; 6 Ottawa Hospital Research Institute, Ottawa, Ontario, Canada; 7 Department of Pathology, University Health Network, Toronto, Ontario, Canada; University of North Carolina School of Medicine, United States of America

## Abstract

Single nucleotide polymorphisms (SNPs) are the most common form of genetic variation. We previously demonstrated that SNPs (rs1800734, rs749072, and rs13098279) in the *MLH1* gene region are associated with *MLH1* promoter island methylation, loss of MLH1 protein expression, and microsatellite instability (MSI) in colorectal cancer (CRC) patients. Recent studies have identified less CpG-dense “shore” regions flanking many CpG islands. These shores often exhibit distinct methylation profiles between different tissues and matched normal versus tumor cells of patients. To date, most epigenetic studies have focused on *somatic* methylation events occurring within solid tumors; less is known of the contributions of peripheral blood cell (PBC) methylation to processes such as aging and tumorigenesis. To address whether *MLH1* methylation in PBCs is correlated with tumorigenesis we utilized the Illumina 450 K microarrays to measure methylation in PBC DNA of 846 healthy controls and 252 CRC patients from Ontario, Canada. Analysis of a region of chromosome 3p21 spanning the *MLH1* locus in healthy controls revealed that a CpG island shore 1 kb upstream of the *MLH1* gene exhibits different methylation profiles when stratified by SNP genotypes (rs1800734, rs749072, and rs13098279). Individuals with wild-type genotypes incur significantly higher PBC shore methylation than heterozygous or homozygous variant carriers (p<1.1×10^−6^; ANOVA). This trend is also seen in CRC cases (p<0.096; ANOVA). Shore methylation also decreases significantly with increasing age in cases and controls. This is the first study of its kind to integrate PBC methylation at a CpG island shore with SNP genotype status in CRC cases and controls. These results indicate that CpG island shore methylation in PBCs may be influenced by genotype as well as the normal aging process.

## Introduction

Epigenetic mechanisms induce functionally relevant changes to the genome without changing the nucleotide sequence itself. These mechanisms include DNA methylation, histone modifications and non-coding RNAs. Of these, DNA methylation is the most studied epigenetic mark, with clear links to a variety of diseases established. In healthy individuals, genome-wide methylation levels are generally elevated at intergenic regions and repetitive sequences (eg. ALU, LINE-1 repeats) while methylation is low or non-existent in the promoter CpG islands of most genes. These methylation patterns reverse with increasing age, as well as in disease states, including cancer [1]–[3]. CpG islands, the sites of age- and cancer-specific epigenetic changes, are defined by a length of at least 200 base pairs containing a GC percentage greater than 50%, and an observed/expected CpG ratio over 0.60 [4]. Recent studies suggest that many CpG islands are flanked by CpG island “shores” which are less dense in CpG content than islands. Nonetheless, shores exhibit more readily distinguishable methylation levels than islands between different tissues as well as between cancer and matched normal DNA [5]. The vast majority of epigenetic studies have investigated methylation at CpG islands; however, the role of CpG island shore methylation is only just beginning to be understood.

The majority of published studies have investigated DNA methylation changes occurring at the tissue level in normal and diseased states, while less is known about methylation occurring in peripheral blood cells (PBCs). Since blood samples are collected easily from patients, and can be measured at multiple time points during disease progression, studying DNA methylation changes in PBCs can potentially be used as a biomarker for various disease outcomes. Utilizing blood samples also allows comparison between healthy controls with diseased patients. Using PBCs as an alternate biological source has potential which requires further systematic investigation, such as integrating PBC methylation with knowledge of the genetic and epigenetic landscape of tissue DNA.

Single nucleotide polymorphisms, or SNPs, are the most common form of genetic variation, with upwards of 3 million SNPs characterized in the human genome by HapMap phase II [6]. Many SNPs have apparently benign phenotypic consequences, while others may predispose to various diseases such as colorectal cancer (CRC) [7]. The underlying mechanism of action of these SNP variants is not always understood. Recently, we demonstrated that certain SNPs in the *mutL homolog 1, colon cancer, nonpolyposis type 2 (E. coli) (MLH1)* gene region are associated with *MLH1* promoter CpG island methylation, loss of MLH1 protein expression, and tumour microsatellite instability (MSI) phenotype in CRC patients [8]. *MLH1* is a key member of a group of DNA mismatch repair (MMR) genes [9]. Function of *MLH1* is lost in a subset of CRC tumours, due to its inactivation through mutation or methylation. This leads to genome-wide accumulation of copy number alterations at short tandem repeats, or microsatellites, termed microsatellite instability (MSI). Approximately 15% of sporadic CRCs exhibit MSI and the majority of these occur due to promoter CpG island methylation of the *MLH1* gene in colon tumors [9], [10].

In previous studies, we examined 102 SNPs spanning 500 kb surrounding the *MLH1* locus [8], [11]. Among these, we observed three SNPs significantly associated with *MLH1* methylation and tumour MSI, which were in strong linkage disequilibrium spanning 197 kb of the genomic region on chromosome 3 which includes *MLH1,* thus constituting a haplotype block at this region. These 3 SNPs include rs1800734 located 93 base pairs upstream of the *MLH1* start site, and rs749072 and rs13098279 which are located further downstream of *MLH1*. We have also shown through *in vitro* studies in transformed colon cancer cell lines that the allelic variant of rs1800734 decreases *MLH1* promoter CpG island-mediated transcriptional activity, thereby providing insight into its potential role as a functional SNP [12].

Taken together, we have demonstrated a link between these SNPs and *MLH1* CpG island methylation in CRC tumours, but the potential correlation of these three SNPs with *MLH1* shore methylation has never been investigated, nor has it been analyzed in peripheral blood cells of normal healthy individuals. Since SNPs are static germline alterations, their potential modifier effects on methylation may be exerted with varying capacity on all tissues of the body, including PBCs from patients. Thus, the goal of our study was to examine the relationship between the three aforementioned *MLH1-*region SNPs and the methylation status of the *MLH1* CpG island shore in PBCs obtained from a cohort of 1,100 population-based healthy controls and CRC patients.

## Materials and Methods

### Ethics Statement

Blood and tissue samples from CRC cases and controls were obtained with informed written consent, following protocols approved by the research ethics board of Mount Sinai Hospital and the University of Toronto.

### Study Subjects

Study participants were recruited through the Ontario Familial Colorectal Cancer Registry (OFCCR), one of six participating cancer registries which are part of the Colon Cancer Family Registry, a US National Cancer Institute-supported consortium. Both primary CRC cases and unaffected controls were accrued through population-based recruitment methods. A detailed account of patient accrual, data collection, and biological specimen collection has been previously described [13], [14]. Briefly, population control subjects were recruited via randomly selected residential telephone numbers in 1999–2000, and by population-based Tax Assessment Rolls of the provincial government, allowing the identification of age- and sex-matched controls. Due to the high proportion of self-reported Caucasians, patients with non-white, unknown or mixed ethnic backgrounds were excluded. Of 2,736 individuals who agreed to participate, 1,336 controls completed family, personal, and diet questionnaires, provided blood samples, and were self-reported as Caucasian. Ontario residents diagnosed with primary CRC from June 1, 1997 to June 30, 2000 between the ages of 20 and 74 were eligible for recruitment to the OFCCR. Cases of familial adenomatous polyposis were excluded from the study and no related cases were used. A total of 1,257 case patients remained after exclusion.

### Single Nucleotide Polymorphism Genotyping

The SNPs chosen for study were selected based on extensive database and literature searches of polymorphisms present on the Affymetrix GeneChip Human Mapping 100 K and 500 K platforms. rs749072 and rs13098279 were chosen because these SNPs are in strong linkage disequilibrium with rs1800734 as well as each other (r^2^>0.73 and D’>0.98). These 3 SNPs are all in Hardy-Weinberg equilibrium (p<10^−4^) [8].

SNP genotyping was performed as described previously [8]. Briefly, peripheral blood cells (PBCs) were isolated from the blood samples provided by CRC cases and controls using Ficoll-Paque gradient centrifugation according to manufacturer’s protocol (Amersham Biosciences, Baie d’Urfé, Quebec, Canada). Genomic DNA was extracted from PBCs by phenol-chloroform or Qiagen DNA extraction kit (Qiagen Inc., Montgomery Co., MD). The fluorogenic 5′ nuclease polymerase chain reaction (PCR) assay was used to genotype rs1800734. This SNP was also genotyped using the Affymetrix GeneChip Human Mapping 100 K and 500 K platforms as part of the Assessment of Risk of Colorectal Tumors in Canada (ARCTIC) project [11] and this data was used as a cross-validation measure. In all, 11 of 1884 (0.58%) samples genotyped gave discordant results between the two platforms. Primer and probe sequences have been described previously [8], [11]. The rs749072 and rs13098279 SNPs were genotyped using the Eurogentec qtPCR kit (Eurogentec, San Diego, CA).

### Methylation Microarray

CpG methylation was measured using Infinium HumanMethylation450 BeadChips from Illumina (San Diego, CA). 998 control samples and 1,103 CRC samples were assayed on 96-well plates; a subset of 65 samples was analyzed in duplicate or triplicate with data available for a total of 136 possible pairs. Bisulphite conversion of DNA was performed using the EZ DNA Methylation-Gold Kit (Zymo Research, Orange, CA). 500 ng of bisulphite converted DNA was used for hybridization to the array following Illumina Infinium HD Methylation Protocol. The efficiency of bisulphite conversion was verified using internal control probes. We excluded from analysis samples that are outliers with respect to internal control probes. Also excluded were CRC cases given chemotherapeutic treatment prior to donation of blood sample, and CRC cases with unknown chemotherapy status. After exclusion, 252 CRC samples and 846 controls remained. The methylation was measured at each CpG site using the fluorescent intensity ratio. After normalization using the internal normalization probes, the resulting value was represented by a β value ranging from 0 (no methylation) to 1 (complete methylation). Values with a detection p-value above 0.01 were removed from analysis.

### Selection of CpG Sites

The Infinium HumanMethylation450 BeadChip captures methylation measurements at over 450,000 CpG sites across the entire genome. We chose every CpG site on chromosome 3 between nucleotide positions 37,018,029 and 37,239,890 (Genome Build 37) spanning a 221 kb region for further analysis. There are 70 CpG sites within this region, encompassing the genes *EPM2A (laforin) interacting protein 1* (*EPM2AIP1), MLH1,* and *leucine rich repeat (in FLII) interacting protein 2* (*LRRFIP2*). The SNPs rs1800734, rs749072, and rs13098279 also occur within this region. This chromosomal region contains a CpG island shore upstream of *MLH1* within the coding region of *EPM2AIP1*. The entire shore spans from nucleotide 37,033,373 to 37,034,166 and contains 13 CpG sites from the array. However, a section of the shore from 37,033,373 to 37,034,166, which exhibited the most significant associations and contains 7 CpG sites, will be the focus of our results.

### Statistics

Methylation was compared between groups using analysis of variance (ANOVA) with a significance level adjusted for multiple comparisons. Groups compared were wild-type, heterozygous, and homozygous variant groups of the three SNP genotypes. Partial correlation was utilized to compare age and methylation, controlling for sex. Gender differences in methylation were tested for association using age at study recruitment as a covariate. Colon cancer diagnosis status was tested for association with percentage methylation for each CpG site. Sex and age at study recruitment were used as covariates. All statistical analysis was performed using SPSS PASW Statistics 18 (Chicago, IL).

## Results

846 controls and 252 CRC cases from the Ontario Familial Colorectal Cancer Registry were successfully analyzed for methylation levels across the genome spanning 450,000 CpG sites. A mean correlation coefficient of 99.45% (range: 95.0–99.9%) was calculated from the comparison of methylation β values between all duplicate pairs. A 221 kb section of DNA from chromosome 3 containing *MLH1* was chosen for statistical analysis based on the presence of 3 SNPs associated with *MLH1* promoter methylation and MSI. Of these, 846 controls and 252 cases were successfully genotyped for rs1800734; 766 controls and 235 cases were genotyped for rs749072 and rs13098279. Clinicopathological characteristics of the population used in this study are shown in Table 1 along with genotypic frequencies for the SNPs rs1800734, rs749072, and rs13098279 for cases and controls. There were no differences in SNP genotype frequencies between different genders (Fisher’s exact test, p = 0.058 for rs1800734, p = 0.074 for rs749072, p = 0.081 for rs13098279) or any associations between genotype and age (ANOVA, p = 0.475 for rs1800734, p = 0.637 for rs749072, p = 0.577 for rs13098279). Results for the seven CpG sites in the *MLH1* CpG island shore are discussed in the text, while results for the entire 70 CpG sites analysed are shown in supplemental files.

**Table 1:** Characteristics of study population.

	CRC Cases	Controls
Characteristic	N (%)	N (%)
Female	122 (48.4)	356 (42.1)
Male	130 (51.6)	490 (57.9)
Age (in years) – mean(SD)	63.4 (8.4)/64.1 (8.3)	64.3 (8.2)
rs1800734 genotype		
Homozygous wild-type (GG)	150 (59.5)	528 (62.5)
Heterozygous (GA)	96 (38.1)	264 (31.2)
Homozygous variant (AA)	6 (2.4)	53 (6.3)
rs749072 genotype		
Homozygous wild-type (AA)	122 (51.9)	438 (51.8)
Heterozygous (AG)	104 (44.3)	271 (32.0)
Homozygous variant (GG)	9 (3.8)	57 (6.7)
rs13098279 genotype		
Homozygous wild-type (GG)	147 (62.6)	491 (64.1)
Heterozygous (GA)	84 (35.7)	233 (27.5)
Homozygous variant (AA)	4 (1.7)	42 (5.5)

Distribution of clinicopathological features in primary colorectal carcinomas and controls from Ontario. Age at study recruitment is indicated for CRC cases and controls. Blood was drawn an average of less than one year and no more than six years after study recruitment.

SD = standard deviation.

### PBC Methylation Differences among SNP Genotypes

We compared methylation in the *MLH1* shore region between different SNP genotypes of rs1800734, rs749072, and rs13098279 in healthy individuals. The mean methylation for each SNP genotype (wild-type, heterozygous, or homozygous variant) was compared using ANOVA at 70 CpG sites. The results for these sites from position 37,018,029 to 37,239,890 on chromosome 3 are shown in Table S1. The results of this analysis for the *MLH1* CpG island shore are shown in Table 2. There are seven CpG sites in the shore region of interest, henceforth to be referred to as sites S1 through S7. The mean methylation in the *MLH1* shore among individuals stratified by SNP genotypes was highest among the wild-type genotype (GG) for rs1800734. The heterozygous genotype (GA) had intermediate levels of methylation while the homozygous variant allele (AA) had the lowest methylation. These differences in methylation among genotypes were statistically significant for all 7 CpG sites localized to the *MLH1* shore region. For example, at S1 mean wild-type methylation was 0.648, heterozygous was 0.607, and homozygous variant methylation was 0.569 (p = 1.93×10^−16^). Similar results were obtained for rs749072 and rs13098279. At S1 rs749072 mean wild-type methylation was 0.647, heterozygous methylation was 0.614, and homozygous variant methylation was 0.578 (p = 7.92×10^−12^). For rs13098270 at S1 mean wild-type methylation was 0.648, heterozygous methylation was 0.606, and homogyzous variant methylation was 0.558 (p = 6.11×10^−17^).

**Table 2:** Methylation between SNP genotypes in healthy controls by ANOVA.

Chromosome 3 Location	Probe ID^a^	CpG Site	Wild-type mean β value (SD)	Heterozygote mean β value (SD)	Homozygote variant mean β value (SD)	P-value
rs1800734			n = 528	n = 264	n = 53	
37,033,373	cg02103401	S1	0.644 (0.080)	0.607 (0.086)	0.567 (0.088)	**5.99×10^−18^**
37, 033,625	cg24607398	S2	0.786 (0.055)	0.758 (0.060)	0.740 (0.063)	**3.91×10^−17^**
37,033,632	cg10990993	S3	0.757 (0.051)	0.728 (0.056)	0.708 (0.054)	**3.50×10^−21^**
37,033,791	cg04726821	S4	0.255 (0.050)	0.229 (0.048)	0.204 (0.046)	**1.51×10^−22^**
37,033,894	cg11291081	S5	0.125 (0.035)	0.117 (0.031)	0.106 (0.029)	**1.11×10^−06^**
37,033,903	cg05670953	S6	0.210 (0.053)	0.194 (0.051)	0.176 (0.047)	**5.88×10^−09^**
37,033,980	cg18320188	S7	0.124 (0.021)	0.118 (0.020)	0.113 (0.019)	**7.31×10^−07^**
**rs749072**			**n = 438**	**n = 271**	**n = 57**	
37,033,373	cg02103401	S1	0.644 (0.082)	0.615 (0.085)	0.579 (0.086)	**1.20×10^−11^**
37, 033,625	cg24607398	S2	0.786 (0.056)	0.763 (0.059)	0.746 (0.057)	**3.67×10^−12^**
37,033,632	cg10990993	S3	0.755 (0.051)	0.735 (0.057)	0.717 (0.056)	**2.53×10^−11^**
37,033,791	cg04726821	S4	0.254 (0.051)	0.233 (0.049)	0.213 (0.048)	**3.92×10^−14^**
37,033,894	cg11291081	S5	0.125 (0.035)	0.118 (0.031)	0.110 (0.031)	**1.29×10^−04^**
37,033,903	cg05670953	S6	0.209 (0.054)	0.197 (0.051)	0.181 (0.049)	**8.79×10^−06^**
37,033,980	cg18320188	S7	0.124 (0.022)	0.118 (0.019)	0.112 (0.019)	**2.20×10^−07^**
**rs13098279**			**n = 491**	**n = 233**	**n = 42**	
37,033,373	cg02103401	S1	0.644 (0.081)	0.607 (0.084)	0.557 (0.086)	**3.04×10^−17^**
37, 033,625	cg24607398	S2	0.785 (0.055)	0.758 (0.059)	0.735 (0.059)	**6.43×10^−16^**
37,033,632	cg10990993	S3	0.756 (0.051)	0.729 (0.055)	0.705 (0.056)	**2.32×10^−18^**
37,033,791	cg04726821	S4	0.254 (0.051)	0.228 (0.047)	0.204 (0.048)	**3.82×10^−20^**
37,033,894	cg11291081	S5	0.124 (0.034)	0.117 (0.031)	0.106 (0.030)	**2.84×10^−05^**
37,033,903	cg05670953	S6	0.209 (0.053)	0.194 (0.052)	0.171 (0.047)	**6.98×10^−08^**
37,033,980	cg18320188	S7	0.124 (0.021)	0.118 (0.019)	0.111 (0.020)	**2.92×10^−07^**

Mean β value of each genotype of the SNPs rs1800734, rs749072, and rs13098279 in healthy controls from Ontario at seven sites in the *MLH1* CpG island shore. Chromosome 3 locations and Probe IDs are the same for CpG sites S1–S7 in subsequent tables. Significant results are bolded when p<0.001.

a Probe ID according to Illumina Infinium HumanMethylation450 array, used throughout in tables.

CI = confidence interval.

We also performed the same analysis for CRC cases, shown in Table 3 for the *MLH1* shore region. The results for all 70 CpG sites are shown in Table S2. However, some CRC cases had undergone chemotherapy prior to providing blood samples for this study (n = 292) and other cases had unknown chemotherapy status (n = 347). This left 252 CRC cases remaining who had definitively not received chemotherapy prior to blood donation. To ensure that chemotherapy does not add a confounding factor to our analyses, we only included cases that had not been given chemotherapy. Stratifying these remaining 252 CRC cases by SNP genotype, the same pattern was found as in controls: those individuals with wild-type genotypes incur higher methylation than those with any other genotype. For example, for rs1800734 at S1 in CRC cases, wild-type methylation was 0.631, heterozygous methylation was 0.606, and homozygous variant methylation was 0.550 (p = 0.01). Comparable significant results were found for rs749072 and rs13098279. Some, but not all, of the *MLH1* shore CpG sites show a significant association with SNP genotype. This is likely due to the smaller sample size of only 252 cases, compared to the 846 controls utilized in a similar analysis.

**Table 3:** Methylation between SNP genotypes in CRC cases by ANOVA.

CpG Site	Wild-type mean β value (SD)	Heterozygote mean β value (SD)	Homozygote variant mean β value (SD)	P-value
rs1800734	n = 150	n = 96	n = 6	
S1	0.631	0.606	0.550	0.010
S2	0.781	0.755	0.729	**6.77×10^−04^**
S3	0.746	0.725	0.716	0.006
S4	0.251	0.222	0.200	**2.06×10^−05^**
S5	0.125	0.114	0.118	0.096
S6	0.206	0.189	0.186	0.060
S7	0.124	0.116	0.120	0.028
**rs749072**	**n = 122**	**n = 103**	**n = 9**	
S1	0.630	0.617	0.590	0.282
S2	0.783	0.762	0.745	0.008
S3	0.747	0.732	0.716	0.042
S4	0.251	0.228	0.207	**6.64×10^−04^**
S5	0.126	0.116	0.106	0.059
S6	0.207	0.190	0.190	0.078
S7	0.125	0.116	0.111	0.007
**rs13098279**	**n = 147**	**n = 84**	**n = 4**	
S1	0.631	0.612	0.550	0.060
S2	0.781	0.758	0.746	0.010
S3	0.747	0.728	0.708	0.019
S4	0.250	0.222	0.189	**9.65×10^−05^**
S5	0.125	0.114	0.118	0.150
S6	0.206	0.189	0.170	0.047
S7	0.124	0.116	0.113	0.020

Mean β value of each genotype of the SNPs rs1800734, rs749072, and rs13098279 in CRC patients from Ontario at seven sites in the *MLH1* CpG island shore. Significant results are bolded when p<0.001.

### Age-related Decrease in Methylation at the *MLH1* Shore Region

Normally, as individuals age, global hypomethylation of the genome occurs combined with increases in methylation at specific genes [3]. To investigate whether the *MLH1* shore region exhibits age-associated changes in methylation, correlation analysis was performed, controlling for sex, shown in Table 4. Results for all 70 CpG sites analyzed are shown in Table S3. This was done in cases and controls separately to confirm whether any age-associated changes in methylation at the shore were exclusive to CRC, or whether they occur in all individuals. There is a trend towards decreasing methylation with increasing age in both cases and controls. In controls at sites S4 and S6 in the *MLH1* shore region, there was a significant decrease in methylation with age. For example, at S4, R = −0.170 (p = 1.30×10^−6^). Similarly, methylation also decreases with increasing age among our case population, significantly so at site S6 [R = −0.236 (p = 4.00×10^−4^)].

**Table 4:** Correlation between age and methylation.

CpG Site	Controls R	P-value	CRC Cases R	P-value
S1	−0.081	0.021	0.042	0.536
S2	−0.085	0.016	−0.037	0.582
S3	−0.087	0.014	−0.120	0.074
S4	−0.170	**1.30×10^−06^**	−0.209	0.002
S5	−0.101	0.004	−0.115	0.089
S6	−0.200	**1.16×10^−08^**	−0.236	**4.00×10^−04^**
S7	−0.007	0.846	0.017	0.797

Partial correlation, controlling for sex, between age and methylation at seven sites in the *MLH1* CpG island shore for CRC cases and controls. Significant results are bolded when p<0.001.

### PBC Methylation Differences among Males and Females

Previous studies have demonstrated that *MLH1* tumor methylation is more prevalent among female, MSI positive CRC patients. Therefore, we compared *MLH1* methylation levels in PBCs between males and females to determine whether gender plays a role in this regard. The results for the *MLH1* shore are found in Table 5, and for all 70 CpG sites analyzed in Table S4. We tested for association using binomial logistic regression using age as a covariate in all cases and controls. For most CpG sites, there are no significant differences in methylation between genders. At S5 and S6, methylation in females is significantly higher than in males (S5: 0.126 vs. 0.118; S6: 0.214 vs. 0.195). For S5, p = 7.05×10^−04^, 95% CI: 0.939 (0.905–0.974); for S6, p = 2.94×10^−04^, 95% CI: 0.939 (0.917–0.962).

**Table 5:** Associations between gender and methylation by logistic regression.

CpG Site	Male Mean β Value (SD) (n = 617)	Female Mean β Value (SD) (n = 476)	P-value	Effect Size	Lower 95% CI	Upper 95% CI
S1	0.623 (0.083)	0.632 (0.087)	0.058	1.014	1.000	1.028
S2	0.773 (0.060)	0.775 (0.059)	0.433	1.008	0.988	1.029
S3	0.745 (0.053)	0.745 (0.056)	0.624	1.006	0.984	1.028
S4	0.244 (0.051)	0.244 (0.053)	0.680	1.005	0.982	1.029
S5	0.126 (0.036)	0.118 (0.032)	0.001	0.939	0.905	0.974
S6	0.214 (0.055)	0.195 (0.050)	0.003	0.939	0.917	0.962
S7	0.123(0.022)	0.120 (0.020)	0.030	0.938	0.885	0.994

Mean β value of is shown for males and females along with logistic regression analysis at seven CpG sites in the *MLH1* CpG island shore. Analysis of male versus female methylation is adjusted for age. Significant results are bolded when p<0.001.

### PBC Methylation Differences among CRC Cases and Controls

**Figure 1:**
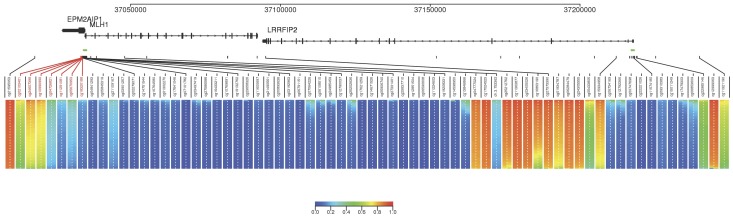
Locations of CpG sites and methylation between cases and controls. Pictured are the 70 CpG sites analyzed, with indicated chromosomal positions located on chromosome 3. The CpG sites are located within the *EPM2AIP1, MLH1,* and *LRRFIP2* genes, with gene exons and transcriptional directions indicated. CpG islands are indicated in green. The seven CpG sites of the *MLH1* shore are highlighted in red. Each vertical bar represents a CpG site, with control methylation, n = 846, displayed to the left and CRC case methylation, n = 252, displayed to the right of the white dotted line. Controls and CRC case samples are displayed layered horizontally from highest methylation to lowest methylation. The distribution of degree of methylation in cases and controls is represented by the colour variation, according to the scale.

We compared methylation in the *MLH1* gene region between CRC patients and healthy controls in 253 cases and 845 controls. A visual representation of case and control methylation at each of the 70 sites analyzed is shown in Figure 1. We tested for association between methylation level and presence of CRC (vs. controls), utilizing sex and age as covariates by binomial logistic regression. The results of this analysis and mean methylation at each CpG site for the *MLH1* CpG island shore in cases and controls are shown in Table 6, and for all 70 CpG sites in Table S5. Though mean methylation in controls is higher than in cases, there is no significant association found between methylation and healthy or diseased state.

**Table 6:** Logistic regression analysis for association with methylation between CRC cases and controls.

CpG Site	Control Mean β Value(SD) (n = 846)	Case Mean β Value (SD) (n = 252)	P-value	Effect Size	Lower 95% CI	Upper 95% CI
S1	0.630 (0.085)	0.620 (0.086)	0.109	1.014	0.997	1.031
S2	0.775 (0.059)	0.770 (0.059)	0.182	1.016	0.992	1.041
S3	0.747 (0.055)	0.738 (0.055)	0.017	1.032	1.006	1.059
S4	0.245 (0.051)	0.238 (0.053)	0.040	1.031	1.001	1.06
S5	0.122 (0.032)	0.121 (0.038)	0.464	1.016	0.973	1.061
S6	0.204 (0.052)	0.199 (0.057)	0.058	1.028	0.999	1.057
S7	0.121 (0.020)	0.121 (0.023)	0.768	1.01	0.943	1.082

Mean β value of CRC cases and controls is shown along with logistic regression analysis at seven CpG sites in the *MLH1* CpG island shore. Analysis of CRC cases versus controls is adjusted for age and sex. Effect size represents the increased risk of CRC per 1% reduction in methylation.

### No Association between MSI Status and Methylation

Methylation of the *MLH1* promoter CpG island is a common occurrence in tumor tissue in MSI CRC [10]. We found no association between tumor MSI status and methylation at either the *MLH1* CpG island or shore in PBC DNA of CRC cases, when tested using binomial logistic regression with age and sex as covariates (data not shown).

### Methylation Levels of the *MLH1* CpG Island and Shore in PBCs

The promoter of *MLH1* spans from chromosome 3 nucleotide position 37,034,130 to 37,034,856 (−711 to +15 relative to the *MLH1* transcriptional start site) [15]. We investigated the methylation status of this promoter island in our PBC samples. The Illumina Infinium HumanMethylation450 microarrays contain 16 CpG sites located within the *MLH1* promoter. We found that overall, methylation is very low among both cases and controls in PBCs, it does not differ significantly when stratified by SNP genotypes, and is not significantly correlated with age. The mean methylation for the CpG sites ranges from 0.004 to 0.064. Differences in methylation among cases, controls, and SNP genotypes and correlations with age can be found for the promoter CpG island region in Tables S1, S2, S3, S4.

CpG island shores can flank CpG islands of genes, being located upstream and/or downstream. In addition to the shore located upstream of the promoter CpG island, which is the focus of this investigation, *MLH1* also has a shore downstream of its island. There are only two CpG sites on the Illumina microarrays which interrogated methylation at this region, at 37,035,399 and 37,036,726. Results, though not significant, for this methylation at this downstream shore can be found in Tables S1, S2, S3, S4.

## Discussion

In this study, we measured methylation in PBC DNA of a large series of healthy individuals, as well as CRC cases, using the Illumina Infinium HumanMethylation450 arrays. We integrated this methylation data with SNP profiling data previously generated by our group for the same controls and cases [8] and found novel, significant associations at the *MLH1* CpG island shore. We have demonstrated that differences in *MLH1* shore region methylation among PBCs are significantly associated with distinct genotypic variants in the *MLH1* gene region. Specifically, a CpG island shore 1 kb upstream of the *MLH1* start site exhibits associations between methylation in PBC DNA in controls with wild-type genotypes of SNPs located over 1 kb away (rs1800734, rs749072) and up to 200 kb away (rs13098279) from this shore region. The variant alleles of these three SNPs are associated with reduced methylation at CpG sites within the *MLH1* shore, significantly lower than either the heterozygous or homozygous wild-type alleles in PBCs. Results also show that methylation of this shore decreases with age, in healthy individuals and CRC cases. Such associations between PBC methylation and genetic variants in a shore region have until now not been described.

Though the concept of CpG islands dates back to the 1980s [16], CpG island shores are a newer element of methylation phenomena that has emerged in recent years [5]. Shores are regions of the genome that flank some CpG islands and have a lower GC content than islands do. Despite this distance from genes and decreased CpG content, methylation of CpG island shores are reported to display more specificity between different tissues, and between normal and cancerous cells from the same patients [5]. Gene expression is also strongly related to shore methylation [5], [17]. In genome-wide methylation analysis, over 50% of the differentially methylated regions between normal colon tissue and tumor tissue were located in shores, rather than islands [5]. Shore methylation has also been shown to discriminate between benign and malignant peripheral nerve sheath tumors [17]. Recent studies have demonstrated that shore methylation decreases with increasing age, concomitantly with global hypomethylation [18]. This is consistent with our results, which showed a decrease in methylation with increasing age at the *MLH1* shore. Though much remains to be discovered about the importance and regulation of shores, methylation at these regions shows potential at discriminating among different tissues, between normal and diseased states, different genotypes, and age.

Earlier studies have shown that DNA sequence can affect methylation at nearby loci [19], [20], as we have demonstrated in our results. More recently it was verified that SNP-dependent DNA methylation alterations can also play a role in disease [21], [22]. We previously reported a significant association between the *MLH1* promoter SNP (rs1800734) and MSI CRCs, and subsequently showed this association being mediated via *MLH1* promoter hypermethylation and loss of MLH1 protein expression contributing to MSI CRC tumors [8], [11]. We further assessed the role of this variant by measuring transcriptional activity of the *MLH1* promoter CpG island of transformed colon cancer cell lines. Cells possessing the variant allele of rs1800734 exhibited decreased transcription compared to wild-type [12]. Though we did not find that rs1800734 increased the overall risk of CRC, only the risk of the MSI phenotype of CRC, a subsequent meta-analysis was performed by another group, which included our data in the analysis. It was found that indeed, the variant allele of this SNP is a modest but significant risk factor for CRC overall, with an odds ratio (95% confidence interval) of 1.06 (1.00–1.11; p = 0.037) [22]. Though we did not find any associations between PBC shore methylation and CRC status, we have clearly demonstrated that these *MLH1-*region SNPs show a strikingly significant association with shore methylation in the peripheral blood of healthy individuals. Perhaps this variant-associated hypomethylation alone does not cause cancer, but in combination with other genetic, epigenetic, and environmental alterations of an individual, it may serve as a low-penetrance susceptibility marker.

Alternatively, there is a possibility that the SNPs rs1800734, rs749072, and rs13098279 are actually linked to a different rare functional variant which is causing these outcomes. Though there is currently no known rare variant in the *MLH1* SNP haplotype block, other studies have analyzed chromosomal regions linked to disease in order to determine the underlying causative variants. For example, microsatellite fine mapping in an affected family determined that a 1.3 Mbp interval of chromosome 1 contained a rare mutation in the gene *UbiA prenyltransferase domain containing 1,* the cause behind Schnyder crystalline corneal dystrophy [23]. Another possibility is that our SNPs serve a currently unknown function. For example, the 8q24 susceptibility locus for breast, prostate, and colorectal cancers [14], [24] contains several SNPs with functional consequences. Rs378854 variant reduces binding of the YY1 transcription factor, leading to increased expression of Pvt1 in prostate cancer cell lines [25] while rs6983267 affects binding of the transcription factor TCF4 in CRC cells [26]. Any function of our SNPs or linkage to another variant is currently unknown, however, and warrants further investigation.

One caveat concerning our results is the inability to ascribe our measured PBC methylation to a specific blood cell type. Peripheral blood consists of natural killer cells, B cells, T cells, monocytes, and granulocytes, each with their own epigenetic profiles. Genome-wide methylation measurements using Illumina 27 K arrays have highlighted regions differentially methylated between different peripheral blood cell populations [27]. Also, peripheral blood subpopulations change with increasing age [28], [29]. Thus, we cannot say for certain whether the methylation changes we see at the *MLH1* shore are present in all PBC types, or perhaps just in a certain subpopulation of the cells, which may also be affected by age. Perhaps the variant-associated hypomethylation we see is particularly pronounced in some PBC types but not others. What we do know is that overall in PBC samples, regardless of cell populations, there are noticeable significant changes in methylation at the *MLH1* shore region.

Overall, this study has numerous strengths. Our large sample size offers high statistical power utilizing both CRC cases and controls. With more than 800 control samples we were able to distinguish differences in methylation based on age and stratified by SNP genotype. Patient and control clinicopathological features have been extensively characterized, as has the epigenetic and genetic features of the *MLH1* gene region. We have now further described the epigenomic landscape of *MLH1* by assessing methylation at its CpG island shore. Our study also benefits from the use of PBC DNA. Blood is an easily accessible biological patient material which can offer information about permanent changes such as germline genetic alterations (SNPs) as well as the varying epigenetic changes resulting in response to both genetic and environmental sources. We have found associations in healthy controls with age and SNP genotype in PBCs. What remains to be seen is whether these patterns exist in other tissues, such as the normal colon, and colon tumour tissue. Additional work for the future includes further analyzing our data garnered from the Infinium HumanMethylation450 BeadChips, arrays which offer comprehensive genome-wide methylation analysis at nearly half a million CpG sites. Thus far we have studied a small region of the genome and found exciting associations. Further probing of the methylomes of our CRC cases and controls may reveal other genomic regions with detectable differences in methylation between cancer and control, SNP variants, gender, age, tumor subtype, and other variables.

In summary, this novel study has demonstrated associations between SNP variants at 3p21 with methylation at a CpG island shore of *MLH1* in peripheral blood cells of 1,100 population-based controls and CRC patients. Our results have also shown an association with decreasing methylation at the shore with age, which may add another facet to potential roles of shore methylation and how it can incur changes based on tissue, presence of cancer, and environment. It is clear that these 3 SNP variants in the *MLH1* region play many roles in colorectal tumorigenesis, including the regulation of *MLH1* methylation at its CpG island shore and island.

## Supporting Information

Table S1 Mean methylation between SNP genotypes for controls.
(DOCX)

Table S2 Mean methylation between SNP genotypes for CRC cases.
(DOCX)

Table S3 Correlation between age and methylation.
(DOCX)

Table S4 Logistic regression analysis for gender.
(DOCX)

Table S5 Regression analysis for CRC cases vs. controls.
(DOCX)
