# Supplementary material for: MLH1 Region Polymorphisms Show a Significant Association with CpG Island Shore Methylation in a Large Cohort of Healthy Individuals
Source: PLoS One. 2012 Dec 11;7(12):e51531. doi: 10.1371/journal.pone.0051531 (PMC3519863; doi:10.1371/journal.pone.0051531)
Supplement: Table S1 — Mean methylation between SNP genotypes for controls. (DOCX) [file pone.0051531.s001.docx]

**Table S1.** Mean methylation between SNP genotypes for controls.

| Shore Site Locations | Chromosome 3 Coordinate | Probe ID | rs1800734 | GG Mean (n=528) | GA Mean (n=264) | AA Mean (n=53) | P-value |
| --- | --- | --- | --- | --- | --- | --- | --- |
|  | 37018029 | cg21595053 |  | 0.932 | 0.933 | 0.933 | 0.743 |
| S1 | 37033373 | cg02103401 |  | 0.648 | 0.607 | 0.569 | 1.93E-16 |
| S2 | 37033625 | cg24607398 |  | 0.787 | 0.759 | 0.741 | 6.59E-14 |
| S3 | 37033632 | cg10990993 |  | 0.760 | 0.730 | 0.707 | 2.15E-19 |
| S4 | 37033791 | cg04726821 |  | 0.256 | 0.232 | 0.205 | 7.24E-18 |
| S5 | 37033894 | cg11291081 |  | 0.125 | 0.118 | 0.105 | 4.42E-06 |
| S6 | 37033903 | cg05670953 |  | 0.211 | 0.196 | 0.175 | 5.80E-08 |
| S7 | 37033980 | cg18320188 |  | 0.124 | 0.119 | 0.112 | 1.33E-05 |
|  | 37034028 | cg04841293 |  | 0.052 | 0.051 | 0.052 | 0.449 |
|  | 37034066 | cg05845319 |  | 0.077 | 0.076 | 0.074 | 0.331 |
|  | 37034084 | cg21109167 |  | 0.177 | 0.169 | 0.165 | 0.007 |
|  | 37034142 | cg03901257 |  | 0.046 | 0.046 | 0.046 | 0.876 |
|  | 37034154 | cg02279071 |  | 0.036 | 0.036 | 0.035 | 0.773 |
|  | 37034166 | cg14751544 |  | 0.064 | 0.063 | 0.062 | 0.522 |
|  | 37034346 | cg16764580 |  | 0.022 | 0.023 | 0.025 | 0.665 |
|  | 37034441 | cg01302270 |  | 0.060 | 0.061 | 0.063 | 0.312 |
|  | 37034473 | cg17641046 |  | 0.063 | 0.064 | 0.065 | 0.383 |
|  | 37034495 | cg07101782 |  | 0.004 | 0.004 | 0.004 | 0.947 |
|  | 37034654 | cg03497419 |  | 0.028 | 0.027 | 0.027 | 0.542 |
|  | 37034661 | cg27586588 |  | 0.034 | 0.035 | 0.034 | 0.659 |
|  | 37034693 | cg16433211 |  | 0.013 | 0.015 | 0.013 | 0.045 |
|  | 37034730 | cg10769891 |  | 0.025 | 0.025 | 0.025 | 0.903 |
|  | 37034739 | cg19132762 |  | 0.018 | 0.019 | 0.018 | 0.920 |
|  | 37034787 | cg23658326 |  | 0.007 | 0.007 | 0.006 | 0.761 |
|  | 37034814 | cg11600697 |  | 0.060 | 0.061 | 0.063 | 0.573 |
|  | 37034825 | cg21490561 |  | 0.039 | 0.039 | 0.037 | 0.505 |
|  | 37034840 | cg00893636 |  | 0.060 | 0.059 | 0.060 | 0.632 |
|  | 37034909 | cg03192963 |  | 0.049 | 0.051 | 0.048 | 0.075 |
|  | 37034956 | cg06791151 |  | 0.015 | 0.015 | 0.015 | 0.612 |
|  | 37034997 | cg07064226 |  | 0.052 | 0.051 | 0.050 | 0.797 |
|  | 37035063 | cg06108510 |  | 0.025 | 0.025 | 0.023 | 0.562 |
|  | 37035090 | cg24985459 |  | 0.002 | 0.002 | 0.002 | 0.823 |
|  | 37035117 | cg12790037 |  | 0.067 | 0.068 | 0.066 | 0.707 |
|  | 37035158 | cg25202636 |  | 0.048 | 0.048 | 0.047 | 0.892 |
|  | 37035168 | cg17621259 |  | 0.006 | 0.006 | 0.006 | 0.479 |
|  | 37035200 | cg14671526 |  | 0.006 | 0.005 | 0.006 | 0.064 |
|  | 37035205 | cg05906740 |  | 0.006 | 0.006 | 0.005 | 0.748 |
|  | 37035207 | cg27331401 |  | 0.059 | 0.061 | 0.060 | 0.172 |
|  | 37035220 | cg25837710 |  | 0.001 | 0.001 | 0.001 | 0.950 |
|  | 37035222 | cg12851504 |  | 0.027 | 0.028 | 0.028 | 0.303 |
|  | 37035228 | cg06590608 |  | 0.006 | 0.006 | 0.006 | 0.902 |
|  | 37035282 | cg11224603 |  | 0.010 | 0.011 | 0.011 | 0.404 |
|  | 37035345 | cg19208331 |  | 0.038 | 0.038 | 0.037 | 0.955 |
|  | 37035355 | cg14598950 |  | 0.024 | 0.025 | 0.025 | 0.792 |
|  | 37035399 | cg13846866 |  | 0.037 | 0.036 | 0.041 | 0.428 |
|  | 37036726 | cg04777024 |  | 0.884 | 0.886 | 0.885 | 0.504 |
|  | 37038591 | cg17024523 |  | 0.912 | 0.913 | 0.915 | 0.494 |
|  | 37048044 | ch.3.753362R |  | 0.146 | 0.145 | 0.141 | 0.463 |
|  | 37055414 | cg25212762 |  | 0.952 | 0.955 | 0.953 | 0.389 |
|  | 37082315 | cg11363877 |  | 0.938 | 0.939 | 0.938 | 0.960 |
|  | 37082380 | cg03405026 |  | 0.927 | 0.927 | 0.926 | 0.789 |
|  | 37092193 | cg16863190 |  | 0.920 | 0.928 | 0.932 | 0.098 |
|  | 37095036 | cg27373390 |  | 0.928 | 0.922 | 0.925 | 0.017 |
|  | 37152029 | cg01934787 |  | 0.920 | 0.917 | 0.926 | 0.271 |
|  | 37173546 | cg06284479 |  | 0.920 | 0.918 | 0.914 | 0.137 |
|  | 37179823 | cg24305555 |  | 0.939 | 0.941 | 0.939 | 0.644 |
|  | 37204814 | cg05433805 |  | 0.525 | 0.512 | 0.497 | 0.050 |
|  | 37212084 | cg15934958 |  | 0.887 | 0.870 | 0.846 | 4.19E-10 |
|  | 37216510 | cg06734169 |  | 0.061 | 0.059 | 0.055 | 0.133 |
|  | 37217087 | cg12792366 |  | 0.045 | 0.043 | 0.041 | 0.276 |
|  | 37217675 | cg00747698 |  | 0.086 | 0.088 | 0.086 | 0.021 |
|  | 37217993 | cg22221026 |  | 0.005 | 0.005 | 0.005 | 0.430 |
|  | 37217996 | cg11574180 |  | 0.036 | 0.037 | 0.037 | 0.375 |
|  | 37218128 | cg09310383 |  | 0.108 | 0.109 | 0.108 | 0.661 |
|  | 37218150 | cg15011249 |  | 0.070 | 0.070 | 0.069 | 0.955 |
|  | 37218212 | cg17479303 |  | 0.040 | 0.038 | 0.041 | 0.277 |
|  | 37218771 | cg06853609 |  | 0.061 | 0.062 | 0.065 | 0.665 |
|  | 37219077 | cg22985146 |  | 0.484 | 0.507 | 0.510 | 6.93E-06 |
|  | 37225266 | cg12999063 |  | 0.946 | 0.932 | 0.907 | 6.54E-07 |
|  | 37239890 | cg11321190 |  | 0.628 | 0.628 | 0.638 | 0.648 |
|  |  |  | rs749072 | TT Mean (n=438) | TC Mean (n=271) | CC Mean (n=57) | 0.743 |
|  | 37018029 | cg21595053 |  | 0.932 | 0.933 | 0.932 | 0.720 |
| S1 | 37033373 | cg02103401 |  | 0.647 | 0.614 | 0.578 | 7.92E-12 |
| S2 | 37033625 | cg24607398 |  | 0.787 | 0.763 | 0.746 | 6.36E-10 |
| S3 | 37033632 | cg10990993 |  | 0.757 | 0.737 | 0.717 | 6.31E-10 |
| S4 | 37033791 | cg04726821 |  | 0.255 | 0.236 | 0.214 | 7.61E-11 |
| S5 | 37033894 | cg11291081 |  | 0.124 | 0.119 | 0.111 | 0.002 |
| S6 | 37033903 | cg05670953 |  | 0.209 | 0.199 | 0.180 | 6.23E-05 |
| S7 | 37033980 | cg18320188 |  | 0.124 | 0.119 | 0.113 | 2.46E-05 |
|  | 37034028 | cg04841293 |  | 0.052 | 0.051 | 0.051 | 0.632 |
|  | 37034066 | cg05845319 |  | 0.077 | 0.076 | 0.076 | 0.513 |
|  | 37034084 | cg21109167 |  | 0.176 | 0.172 | 0.163 | 0.037 |
|  | 37034142 | cg03901257 |  | 0.046 | 0.046 | 0.046 | 0.792 |
|  | 37034154 | cg02279071 |  | 0.037 | 0.036 | 0.036 | 0.652 |
|  | 37034166 | cg14751544 |  | 0.065 | 0.062 | 0.063 | 0.103 |
|  | 37034346 | cg16764580 |  | 0.022 | 0.024 | 0.025 | 0.320 |
|  | 37034441 | cg01302270 |  | 0.060 | 0.061 | 0.061 | 0.828 |
|  | 37034473 | cg17641046 |  | 0.062 | 0.064 | 0.064 | 0.400 |
|  | 37034495 | cg07101782 |  | 0.004 | 0.004 | 0.004 | 0.960 |
|  | 37034654 | cg03497419 |  | 0.028 | 0.026 | 0.027 | 0.478 |
|  | 37034661 | cg27586588 |  | 0.034 | 0.035 | 0.033 | 0.444 |
|  | 37034693 | cg16433211 |  | 0.013 | 0.015 | 0.012 | 0.099 |
|  | 37034730 | cg10769891 |  | 0.025 | 0.025 | 0.025 | 0.566 |
|  | 37034739 | cg19132762 |  | 0.018 | 0.019 | 0.017 | 0.583 |
|  | 37034787 | cg23658326 |  | 0.007 | 0.007 | 0.006 | 0.790 |
|  | 37034814 | cg11600697 |  | 0.060 | 0.061 | 0.060 | 0.779 |
|  | 37034825 | cg21490561 |  | 0.039 | 0.038 | 0.038 | 0.704 |
|  | 37034840 | cg00893636 |  | 0.060 | 0.059 | 0.059 | 0.179 |
|  | 37034909 | cg03192963 |  | 0.049 | 0.051 | 0.048 | 0.028 |
|  | 37034956 | cg06791151 |  | 0.015 | 0.015 | 0.015 | 0.362 |
|  | 37034997 | cg07064226 |  | 0.051 | 0.053 | 0.049 | 0.408 |
|  | 37035063 | cg06108510 |  | 0.025 | 0.025 | 0.022 | 0.217 |
|  | 37035090 | cg24985459 |  | 0.002 | 0.001 | 0.002 | 0.270 |
|  | 37035117 | cg12790037 |  | 0.067 | 0.067 | 0.067 | 0.943 |
|  | 37035158 | cg25202636 |  | 0.048 | 0.047 | 0.049 | 0.808 |
|  | 37035168 | cg17621259 |  | 0.006 | 0.006 | 0.005 | 0.428 |
|  | 37035200 | cg14671526 |  | 0.006 | 0.005 | 0.006 | 0.142 |
|  | 37035205 | cg05906740 |  | 0.006 | 0.006 | 0.005 | 0.611 |
|  | 37035207 | cg27331401 |  | 0.059 | 0.060 | 0.058 | 0.177 |
|  | 37035220 | cg25837710 |  | 0.001 | 0.001 | 0.001 | 0.987 |
|  | 37035222 | cg12851504 |  | 0.027 | 0.028 | 0.028 | 0.730 |
|  | 37035228 | cg06590608 |  | 0.006 | 0.006 | 0.007 | 0.671 |
|  | 37035282 | cg11224603 |  | 0.010 | 0.011 | 0.011 | 0.295 |
|  | 37035345 | cg19208331 |  | 0.038 | 0.038 | 0.037 | 0.677 |
|  | 37035355 | cg14598950 |  | 0.024 | 0.025 | 0.024 | 0.757 |
|  | 37035399 | cg13846866 |  | 0.037 | 0.034 | 0.042 | 0.100 |
|  | 37036726 | cg04777024 |  | 0.884 | 0.887 | 0.884 | 0.173 |
|  | 37038591 | cg17024523 |  | 0.912 | 0.914 | 0.914 | 0.319 |
|  | 37048044 | ch.3.753362R |  | 0.146 | 0.145 | 0.141 | 0.330 |
|  | 37055414 | cg25212762 |  | 0.952 | 0.956 | 0.952 | 0.080 |
|  | 37082315 | cg11363877 |  | 0.938 | 0.939 | 0.940 | 0.593 |
|  | 37082380 | cg03405026 |  | 0.927 | 0.928 | 0.925 | 0.324 |
|  | 37092193 | cg16863190 |  | 0.919 | 0.924 | 0.936 | 0.121 |
|  | 37095036 | cg27373390 |  | 0.927 | 0.924 | 0.928 | 0.187 |
|  | 37152029 | cg01934787 |  | 0.920 | 0.918 | 0.926 | 0.350 |
|  | 37173546 | cg06284479 |  | 0.920 | 0.918 | 0.915 | 0.139 |
|  | 37179823 | cg24305555 |  | 0.939 | 0.942 | 0.935 | 0.053 |
|  | 37204814 | cg05433805 |  | 0.526 | 0.516 | 0.498 | 0.081 |
|  | 37212084 | cg15934958 |  | 0.887 | 0.873 | 0.845 | 7.56E-10 |
|  | 37216510 | cg06734169 |  | 0.060 | 0.060 | 0.055 | 0.335 |
|  | 37217087 | cg12792366 |  | 0.046 | 0.043 | 0.040 | 0.076 |
|  | 37217675 | cg00747698 |  | 0.086 | 0.088 | 0.087 | 0.088 |
|  | 37217993 | cg22221026 |  | 0.006 | 0.005 | 0.005 | 0.140 |
|  | 37217996 | cg11574180 |  | 0.036 | 0.036 | 0.037 | 0.583 |
|  | 37218128 | cg09310383 |  | 0.108 | 0.109 | 0.109 | 0.429 |
|  | 37218150 | cg15011249 |  | 0.070 | 0.069 | 0.069 | 0.492 |
|  | 37218212 | cg17479303 |  | 0.040 | 0.038 | 0.044 | 0.025 |
|  | 37218771 | cg06853609 |  | 0.061 | 0.062 | 0.064 | 0.702 |
|  | 37219077 | cg22985146 |  | 0.481 | 0.505 | 0.510 | 1.68E-06 |
|  | 37225266 | cg12999063 |  | 0.946 | 0.935 | 0.927 | 0.001 |
|  | 37239890 | cg11321190 |  | 0.625 | 0.630 | 0.639 | 0.366 |
|  |  |  | rs130988279 | GG Mean (n=491) | GA Mean (n=233) | AA Mean (n=42) | P-value |
|  | 37018029 | cg21595053 |  | 0.932 | 0.933 | 0.933 | 0.806 |
| S1 | 37033373 | cg02103401 |  | 0.648 | 0.606 | 0.558 | 6.11E-17 |
| S2 | 37033625 | cg24607398 |  | 0.787 | 0.758 | 0.734 | 6.17E-14 |
| S3 | 37033632 | cg10990993 |  | 0.759 | 0.730 | 0.704 | 4.17E-17 |
| S4 | 37033791 | cg04726821 |  | 0.256 | 0.230 | 0.205 | 3.79E-16 |
| S5 | 37033894 | cg11291081 |  | 0.124 | 0.118 | 0.105 | 8.12E-05 |
| S6 | 37033903 | cg05670953 |  | 0.210 | 0.196 | 0.172 | 1.07E-06 |
| S7 | 37033980 | cg18320188 |  | 0.123 | 0.118 | 0.110 | 9.91E-06 |
|  | 37034028 | cg04841293 |  | 0.052 | 0.051 | 0.051 | 0.622 |
|  | 37034066 | cg05845319 |  | 0.077 | 0.075 | 0.074 | 0.416 |
|  | 37034084 | cg21109167 |  | 0.176 | 0.170 | 0.164 | 0.033 |
|  | 37034142 | cg03901257 |  | 0.046 | 0.046 | 0.047 | 0.885 |
|  | 37034154 | cg02279071 |  | 0.036 | 0.036 | 0.035 | 0.763 |
|  | 37034166 | cg14751544 |  | 0.064 | 0.063 | 0.062 | 0.278 |
|  | 37034346 | cg16764580 |  | 0.022 | 0.024 | 0.024 | 0.594 |
|  | 37034441 | cg01302270 |  | 0.060 | 0.061 | 0.063 | 0.295 |
|  | 37034473 | cg17641046 |  | 0.062 | 0.065 | 0.064 | 0.261 |
|  | 37034495 | cg07101782 |  | 0.004 | 0.004 | 0.004 | 0.963 |
|  | 37034654 | cg03497419 |  | 0.028 | 0.027 | 0.028 | 0.656 |
|  | 37034661 | cg27586588 |  | 0.034 | 0.035 | 0.033 | 0.558 |
|  | 37034693 | cg16433211 |  | 0.013 | 0.015 | 0.012 | 0.103 |
|  | 37034730 | cg10769891 |  | 0.025 | 0.025 | 0.024 | 0.759 |
|  | 37034739 | cg19132762 |  | 0.018 | 0.019 | 0.018 | 0.877 |
|  | 37034787 | cg23658326 |  | 0.007 | 0.007 | 0.006 | 0.672 |
|  | 37034814 | cg11600697 |  | 0.060 | 0.061 | 0.061 | 0.512 |
|  | 37034825 | cg21490561 |  | 0.039 | 0.039 | 0.038 | 0.764 |
|  | 37034840 | cg00893636 |  | 0.059 | 0.059 | 0.059 | 0.862 |
|  | 37034909 | cg03192963 |  | 0.049 | 0.051 | 0.048 | 0.073 |
|  | 37034956 | cg06791151 |  | 0.015 | 0.015 | 0.016 | 0.380 |
|  | 37034997 | cg07064226 |  | 0.052 | 0.052 | 0.052 | 0.986 |
|  | 37035063 | cg06108510 |  | 0.025 | 0.024 | 0.022 | 0.232 |
|  | 37035090 | cg24985459 |  | 0.002 | 0.001 | 0.002 | 0.373 |
|  | 37035117 | cg12790037 |  | 0.067 | 0.067 | 0.066 | 0.808 |
|  | 37035158 | cg25202636 |  | 0.048 | 0.048 | 0.047 | 0.948 |
|  | 37035168 | cg17621259 |  | 0.006 | 0.006 | 0.005 | 0.311 |
|  | 37035200 | cg14671526 |  | 0.006 | 0.005 | 0.005 | 0.238 |
|  | 37035205 | cg05906740 |  | 0.006 | 0.006 | 0.005 | 0.715 |
|  | 37035207 | cg27331401 |  | 0.059 | 0.061 | 0.059 | 0.176 |
|  | 37035220 | cg25837710 |  | 0.001 | 0.001 | 0.001 | 0.776 |
|  | 37035222 | cg12851504 |  | 0.027 | 0.028 | 0.028 | 0.353 |
|  | 37035228 | cg06590608 |  | 0.006 | 0.006 | 0.006 | 0.995 |
|  | 37035282 | cg11224603 |  | 0.010 | 0.011 | 0.011 | 0.455 |
|  | 37035345 | cg19208331 |  | 0.038 | 0.038 | 0.037 | 0.918 |
|  | 37035355 | cg14598950 |  | 0.024 | 0.025 | 0.024 | 0.565 |
|  | 37035399 | cg13846866 |  | 0.036 | 0.035 | 0.044 | 0.153 |
|  | 37036726 | cg04777024 |  | 0.884 | 0.887 | 0.884 | 0.313 |
|  | 37038591 | cg17024523 |  | 0.912 | 0.914 | 0.915 | 0.392 |
|  | 37048044 | ch.3.753362R |  | 0.146 | 0.145 | 0.143 | 0.709 |
|  | 37055414 | cg25212762 |  | 0.952 | 0.956 | 0.951 | 0.076 |
|  | 37082315 | cg11363877 |  | 0.938 | 0.939 | 0.940 | 0.822 |
|  | 37082380 | cg03405026 |  | 0.927 | 0.927 | 0.926 | 0.763 |
|  | 37092193 | cg16863190 |  | 0.918 | 0.928 | 0.934 | 0.071 |
|  | 37095036 | cg27373390 |  | 0.928 | 0.923 | 0.926 | 0.149 |
|  | 37152029 | cg01934787 |  | 0.920 | 0.919 | 0.925 | 0.661 |
|  | 37173546 | cg06284479 |  | 0.920 | 0.919 | 0.913 | 0.110 |
|  | 37179823 | cg24305555 |  | 0.939 | 0.941 | 0.937 | 0.374 |
|  | 37204814 | cg05433805 |  | 0.526 | 0.513 | 0.496 | 0.083 |
|  | 37212084 | cg15934958 |  | 0.886 | 0.872 | 0.842 | 1.18E-08 |
|  | 37216510 | cg06734169 |  | 0.060 | 0.059 | 0.058 | 0.685 |
|  | 37217087 | cg12792366 |  | 0.045 | 0.043 | 0.041 | 0.237 |
|  | 37217675 | cg00747698 |  | 0.086 | 0.088 | 0.086 | 0.047 |
|  | 37217993 | cg22221026 |  | 0.006 | 0.005 | 0.005 | 0.293 |
|  | 37217996 | cg11574180 |  | 0.036 | 0.036 | 0.037 | 0.498 |
|  | 37218128 | cg09310383 |  | 0.108 | 0.110 | 0.107 | 0.224 |
|  | 37218150 | cg15011249 |  | 0.070 | 0.069 | 0.069 | 0.841 |
|  | 37218212 | cg17479303 |  | 0.040 | 0.038 | 0.044 | 0.034 |
|  | 37218771 | cg06853609 |  | 0.060 | 0.064 | 0.064 | 0.391 |
|  | 37219077 | cg22985146 |  | 0.483 | 0.506 | 0.512 | 1.02E-05 |
|  | 37225266 | cg12999063 |  | 0.946 | 0.933 | 0.917 | 7.88E-06 |
|  | 37239890 | cg11321190 |  | 0.629 | 0.625 | 0.633 | 0.792 |

Mean β value comparison of each genotype by ANOVA for the SNPs rs1800734, rs749072, and rs13098279 in controls at 70 CpG sites in 3p21-3p22.
