# Supplementary material for: MLH1 Region Polymorphisms Show a Significant Association with CpG Island Shore Methylation in a Large Cohort of Healthy Individuals
Source: PLoS One. 2012 Dec 11;7(12):e51531. doi: 10.1371/journal.pone.0051531 (PMC3519863; doi:10.1371/journal.pone.0051531)
Supplement: Table S2 — Mean methylation between SNP genotypes for CRC cases. (DOCX) [file pone.0051531.s002.docx]

**Table S2.** Mean methylation between SNP genotypes for CRC cases.

| Shore Site Locations | Chromosome 3 Coordinate | Probe ID | rs1800734 | GG Mean (n=150) | GA Mean (n=96) | AA Mean (n=6) | P-value |
| --- | --- | --- | --- | --- | --- | --- | --- |
|  | 37018029 | cg21595053 |  | 0.933 | 0.937 | 0.946 | 0.018 |
| S1 | 37033373 | cg02103401 |  | 0.631 | 0.606 | 0.550 | 0.010 |
| S2 | 37033625 | cg24607398 |  | 0.781 | 0.755 | 0.729 | 6.77E-04 |
| S3 | 37033632 | cg10990993 |  | 0.746 | 0.725 | 0.716 | 0.006 |
| S4 | 37033791 | cg04726821 |  | 0.251 | 0.222 | 0.200 | 2.06E-05 |
| S5 | 37033894 | cg11291081 |  | 0.125 | 0.114 | 0.118 | 0.096 |
| S6 | 37033903 | cg05670953 |  | 0.206 | 0.189 | 0.186 | 0.060 |
| S7 | 37033980 | cg18320188 |  | 0.124 | 0.116 | 0.120 | 0.028 |
|  | 37034028 | cg04841293 |  | 0.050 | 0.050 | 0.053 | 0.673 |
|  | 37034066 | cg05845319 |  | 0.077 | 0.074 | 0.082 | 0.398 |
|  | 37034084 | cg21109167 |  | 0.173 | 0.165 | 0.161 | 0.163 |
|  | 37034142 | cg03901257 |  | 0.045 | 0.042 | 0.047 | 0.185 |
|  | 37034154 | cg02279071 |  | 0.036 | 0.033 | 0.038 | 0.069 |
|  | 37034166 | cg14751544 |  | 0.063 | 0.060 | 0.068 | 0.233 |
|  | 37034346 | cg16764580 |  | 0.026 | 0.027 | 0.038 | 0.442 |
|  | 37034441 | cg01302270 |  | 0.060 | 0.061 | 0.072 | 0.083 |
|  | 37034473 | cg17641046 |  | 0.063 | 0.063 | 0.082 | 0.065 |
|  | 37034495 | cg07101782 |  | 0.004 | 0.004 | 0.013 | 3.24E-07 |
|  | 37034654 | cg03497419 |  | 0.029 | 0.026 | 0.034 | 0.169 |
|  | 37034661 | cg27586588 |  | 0.035 | 0.032 | 0.041 | 0.173 |
|  | 37034693 | cg16433211 |  | 0.012 | 0.012 | 0.013 | 0.945 |
|  | 37034730 | cg10769891 |  | 0.025 | 0.024 | 0.030 | 0.248 |
|  | 37034739 | cg19132762 |  | 0.018 | 0.017 | 0.025 | 0.087 |
|  | 37034787 | cg23658326 |  | 0.008 | 0.007 | 0.008 | 0.762 |
|  | 37034814 | cg11600697 |  | 0.060 | 0.061 | 0.070 | 0.339 |
|  | 37034825 | cg21490561 |  | 0.037 | 0.036 | 0.038 | 0.719 |
|  | 37034840 | cg00893636 |  | 0.060 | 0.060 | 0.064 | 0.503 |
|  | 37034909 | cg03192963 |  | 0.050 | 0.050 | 0.053 | 0.629 |
|  | 37034956 | cg06791151 |  | 0.014 | 0.014 | 0.020 | 0.016 |
|  | 37034997 | cg07064226 |  | 0.049 | 0.053 | 0.061 | 0.150 |
|  | 37035063 | cg06108510 |  | 0.027 | 0.025 | 0.020 | 0.649 |
|  | 37035090 | cg24985459 |  | 0.001 | 0.004 | 0.002 | 0.036 |
|  | 37035117 | cg12790037 |  | 0.067 | 0.065 | 0.072 | 0.149 |
|  | 37035158 | cg25202636 |  | 0.047 | 0.052 | 0.062 | 0.142 |
|  | 37035168 | cg17621259 |  | 0.007 | 0.006 | 0.013 | 0.001 |
|  | 37035200 | cg14671526 |  | 0.007 | 0.007 | 0.013 | 0.151 |
|  | 37035205 | cg05906740 |  | 0.007 | 0.006 | 0.007 | 0.503 |
|  | 37035207 | cg27331401 |  | 0.059 | 0.059 | 0.071 | 0.233 |
|  | 37035220 | cg25837710 |  | 0.001 | 0.001 | 0.007 | 0.001 |
|  | 37035222 | cg12851504 |  | 0.026 | 0.027 | 0.034 | 0.163 |
|  | 37035228 | cg06590608 |  | 0.006 | 0.006 | 0.011 | 0.073 |
|  | 37035282 | cg11224603 |  | 0.012 | 0.012 | 0.016 | 0.409 |
|  | 37035345 | cg19208331 |  | 0.037 | 0.036 | 0.037 | 0.832 |
|  | 37035355 | cg14598950 |  | 0.024 | 0.023 | 0.025 | 0.801 |
|  | 37035399 | cg13846866 |  | 0.038 | 0.038 | 0.056 | 0.405 |
|  | 37036726 | cg04777024 |  | 0.885 | 0.889 | 0.869 | 0.045 |
|  | 37038591 | cg17024523 |  | 0.914 | 0.918 | 0.921 | 0.247 |
|  | 37048044 | ch.3.753362R |  | 0.150 | 0.144 | 0.152 | 0.270 |
|  | 37055414 | cg25212762 |  | 0.951 | 0.952 | 0.959 | 0.772 |
|  | 37082315 | cg11363877 |  | 0.939 | 0.940 | 0.947 | 0.574 |
|  | 37082380 | cg03405026 |  | 0.927 | 0.928 | 0.931 | 0.568 |
|  | 37092193 | cg16863190 |  | 0.916 | 0.901 | 0.878 | 0.393 |
|  | 37095036 | cg27373390 |  | 0.928 | 0.930 | 0.917 | 0.424 |
|  | 37152029 | cg01934787 |  | 0.919 | 0.914 | 0.909 | 0.649 |
|  | 37173546 | cg06284479 |  | 0.917 | 0.921 | 0.925 | 0.214 |
|  | 37179823 | cg24305555 |  | 0.938 | 0.944 | 0.938 | 0.157 |
|  | 37204814 | cg05433805 |  | 0.513 | 0.515 | 0.528 | 0.925 |
|  | 37212084 | cg15934958 |  | 0.885 | 0.869 | 0.890 | 0.086 |
|  | 37216510 | cg06734169 |  | 0.061 | 0.057 | 0.070 | 0.494 |
|  | 37217087 | cg12792366 |  | 0.043 | 0.043 | 0.028 | 0.433 |
|  | 37217675 | cg00747698 |  | 0.088 | 0.085 | 0.091 | 0.113 |
|  | 37217993 | cg22221026 |  | 0.005 | 0.005 | 0.008 | 0.300 |
|  | 37217996 | cg11574180 |  | 0.035 | 0.035 | 0.040 | 0.444 |
|  | 37218128 | cg09310383 |  | 0.110 | 0.110 | 0.118 | 0.292 |
|  | 37218150 | cg15011249 |  | 0.069 | 0.067 | 0.064 | 0.521 |
|  | 37218212 | cg17479303 |  | 0.040 | 0.036 | 0.032 | 0.087 |
|  | 37218771 | cg06853609 |  | 0.062 | 0.059 | 0.041 | 0.289 |
|  | 37219077 | cg22985146 |  | 0.490 | 0.508 | 0.517 | 0.118 |
|  | 37225266 | cg12999063 |  | 0.946 | 0.941 | 0.947 | 0.540 |
|  | 37239890 | cg11321190 |  | 0.610 | 0.618 | 0.625 | 0.773 |
|  |  |  | rs749072 | TT Mean (n=122) | TC Mean (n=103) | CC Mean (n=9) | P-value |
|  | 37018029 | cg21595053 |  | 0.934 | 0.935 | 0.945 | 0.091 |
| S1 | 37033373 | cg02103401 |  | 0.630 | 0.617 | 0.590 | 0.282 |
| S2 | 37033625 | cg24607398 |  | 0.783 | 0.762 | 0.745 | 0.008 |
| S3 | 37033632 | cg10990993 |  | 0.747 | 0.732 | 0.716 | 0.042 |
| S4 | 37033791 | cg04726821 |  | 0.251 | 0.228 | 0.207 | 6.64E-04 |
| S5 | 37033894 | cg11291081 |  | 0.126 | 0.116 | 0.106 | 0.059 |
| S6 | 37033903 | cg05670953 |  | 0.207 | 0.190 | 0.190 | 0.078 |
| S7 | 37033980 | cg18320188 |  | 0.125 | 0.116 | 0.111 | 0.007 |
|  | 37034028 | cg04841293 |  | 0.050 | 0.049 | 0.049 | 0.667 |
|  | 37034066 | cg05845319 |  | 0.076 | 0.073 | 0.077 | 0.329 |
|  | 37034084 | cg21109167 |  | 0.174 | 0.165 | 0.156 | 0.112 |
|  | 37034142 | cg03901257 |  | 0.045 | 0.042 | 0.045 | 0.274 |
|  | 37034154 | cg02279071 |  | 0.035 | 0.033 | 0.033 | 0.346 |
|  | 37034166 | cg14751544 |  | 0.062 | 0.060 | 0.062 | 0.545 |
|  | 37034346 | cg16764580 |  | 0.027 | 0.026 | 0.039 | 0.340 |
|  | 37034441 | cg01302270 |  | 0.060 | 0.061 | 0.068 | 0.260 |
|  | 37034473 | cg17641046 |  | 0.062 | 0.063 | 0.077 | 0.076 |
|  | 37034495 | cg07101782 |  | 0.004 | 0.004 | 0.012 | 3.93E-08 |
|  | 37034654 | cg03497419 |  | 0.029 | 0.027 | 0.025 | 0.420 |
|  | 37034661 | cg27586588 |  | 0.034 | 0.034 | 0.034 | 0.991 |
|  | 37034693 | cg16433211 |  | 0.012 | 0.012 | 0.017 | 0.260 |
|  | 37034730 | cg10769891 |  | 0.025 | 0.024 | 0.028 | 0.489 |
|  | 37034739 | cg19132762 |  | 0.018 | 0.017 | 0.023 | 0.212 |
|  | 37034787 | cg23658326 |  | 0.007 | 0.007 | 0.012 | 0.012 |
|  | 37034814 | cg11600697 |  | 0.059 | 0.061 | 0.070 | 0.190 |
|  | 37034825 | cg21490561 |  | 0.037 | 0.036 | 0.034 | 0.778 |
|  | 37034840 | cg00893636 |  | 0.060 | 0.060 | 0.060 | 0.973 |
|  | 37034909 | cg03192963 |  | 0.050 | 0.050 | 0.051 | 0.929 |
|  | 37034956 | cg06791151 |  | 0.014 | 0.014 | 0.016 | 0.251 |
|  | 37034997 | cg07064226 |  | 0.048 | 0.053 | 0.051 | 0.276 |
|  | 37035063 | cg06108510 |  | 0.026 | 0.026 | 0.019 | 0.561 |
|  | 37035090 | cg24985459 |  | 0.001 | 0.003 | 0.006 | 0.115 |
|  | 37035117 | cg12790037 |  | 0.066 | 0.066 | 0.068 | 0.662 |
|  | 37035158 | cg25202636 |  | 0.045 | 0.051 | 0.055 | 0.167 |
|  | 37035168 | cg17621259 |  | 0.007 | 0.006 | 0.012 | 0.001 |
|  | 37035200 | cg14671526 |  | 0.007 | 0.007 | 0.014 | 0.022 |
|  | 37035205 | cg05906740 |  | 0.007 | 0.006 | 0.009 | 0.330 |
|  | 37035207 | cg27331401 |  | 0.058 | 0.060 | 0.068 | 0.124 |
|  | 37035220 | cg25837710 |  | 0.001 | 0.001 | 0.004 | 0.003 |
|  | 37035222 | cg12851504 |  | 0.026 | 0.027 | 0.032 | 0.124 |
|  | 37035228 | cg06590608 |  | 0.006 | 0.006 | 0.009 | 0.207 |
|  | 37035282 | cg11224603 |  | 0.011 | 0.012 | 0.013 | 0.570 |
|  | 37035345 | cg19208331 |  | 0.036 | 0.038 | 0.036 | 0.785 |
|  | 37035355 | cg14598950 |  | 0.023 | 0.023 | 0.027 | 0.504 |
|  | 37035399 | cg13846866 |  | 0.039 | 0.037 | 0.034 | 0.884 |
|  | 37036726 | cg04777024 |  | 0.886 | 0.888 | 0.884 | 0.796 |
|  | 37038591 | cg17024523 |  | 0.916 | 0.917 | 0.923 | 0.481 |
|  | 37048044 | ch.3.753362R |  | 0.149 | 0.143 | 0.151 | 0.240 |
|  | 37055414 | cg25212762 |  | 0.949 | 0.954 | 0.940 | 0.243 |
|  | 37082315 | cg11363877 |  | 0.940 | 0.939 | 0.941 | 0.898 |
|  | 37082380 | cg03405026 |  | 0.927 | 0.928 | 0.927 | 0.648 |
|  | 37092193 | cg16863190 |  | 0.913 | 0.908 | 0.878 | 0.598 |
|  | 37095036 | cg27373390 |  | 0.929 | 0.929 | 0.922 | 0.683 |
|  | 37152029 | cg01934787 |  | 0.917 | 0.917 | 0.896 | 0.482 |
|  | 37173546 | cg06284479 |  | 0.919 | 0.921 | 0.923 | 0.744 |
|  | 37179823 | cg24305555 |  | 0.939 | 0.943 | 0.943 | 0.417 |
|  | 37204814 | cg05433805 |  | 0.514 | 0.515 | 0.521 | 0.983 |
|  | 37212084 | cg15934958 |  | 0.889 | 0.869 | 0.871 | 0.023 |
|  | 37216510 | cg06734169 |  | 0.061 | 0.057 | 0.059 | 0.723 |
|  | 37217087 | cg12792366 |  | 0.043 | 0.044 | 0.032 | 0.469 |
|  | 37217675 | cg00747698 |  | 0.087 | 0.085 | 0.088 | 0.409 |
|  | 37217993 | cg22221026 |  | 0.005 | 0.005 | 0.006 | 0.927 |
|  | 37217996 | cg11574180 |  | 0.035 | 0.035 | 0.039 | 0.424 |
|  | 37218128 | cg09310383 |  | 0.110 | 0.110 | 0.110 | 0.935 |
|  | 37218150 | cg15011249 |  | 0.068 | 0.068 | 0.065 | 0.733 |
|  | 37218212 | cg17479303 |  | 0.040 | 0.037 | 0.039 | 0.342 |
|  | 37218771 | cg06853609 |  | 0.064 | 0.057 | 0.043 | 0.122 |
|  | 37219077 | cg22985146 |  | 0.490 | 0.504 | 0.507 | 0.298 |
|  | 37225266 | cg12999063 |  | 0.946 | 0.942 | 0.946 | 0.703 |
|  | 37239890 | cg11321190 |  | 0.606 | 0.622 | 0.651 | 0.193 |
|  |  |  | rs13098279 | GG Mean (n=147) | GA Mean (n=84) | AA Mean (n=4) | P-value |
|  | 37018029 | cg21595053 |  | 0.934 | 0.937 | 0.952 | 0.023 |
| S1 | 37033373 | cg02103401 |  | 0.631 | 0.612 | 0.550 | 0.060 |
| S2 | 37033625 | cg24607398 |  | 0.781 | 0.758 | 0.746 | 0.010 |
| S3 | 37033632 | cg10990993 |  | 0.747 | 0.728 | 0.708 | 0.019 |
| S4 | 37033791 | cg04726821 |  | 0.250 | 0.222 | 0.189 | 9.65E-05 |
| S5 | 37033894 | cg11291081 |  | 0.125 | 0.114 | 0.118 | 0.150 |
| S6 | 37033903 | cg05670953 |  | 0.206 | 0.189 | 0.170 | 0.047 |
| S7 | 37033980 | cg18320188 |  | 0.124 | 0.116 | 0.113 | 0.020 |
|  | 37034028 | cg04841293 |  | 0.050 | 0.050 | 0.051 | 0.966 |
|  | 37034066 | cg05845319 |  | 0.076 | 0.074 | 0.074 | 0.743 |
|  | 37034084 | cg21109167 |  | 0.173 | 0.164 | 0.148 | 0.146 |
|  | 37034142 | cg03901257 |  | 0.044 | 0.043 | 0.045 | 0.841 |
|  | 37034154 | cg02279071 |  | 0.035 | 0.032 | 0.039 | 0.225 |
|  | 37034166 | cg14751544 |  | 0.062 | 0.060 | 0.065 | 0.491 |
|  | 37034346 | cg16764580 |  | 0.027 | 0.026 | 0.049 | 0.198 |
|  | 37034441 | cg01302270 |  | 0.060 | 0.061 | 0.073 | 0.177 |
|  | 37034473 | cg17641046 |  | 0.063 | 0.062 | 0.091 | 0.010 |
|  | 37034495 | cg07101782 |  | 0.004 | 0.004 | 0.017 | 1.91E-09 |
|  | 37034654 | cg03497419 |  | 0.028 | 0.026 | 0.030 | 0.530 |
|  | 37034661 | cg27586588 |  | 0.034 | 0.033 | 0.042 | 0.311 |
|  | 37034693 | cg16433211 |  | 0.013 | 0.012 | 0.017 | 0.519 |
|  | 37034730 | cg10769891 |  | 0.025 | 0.024 | 0.031 | 0.283 |
|  | 37034739 | cg19132762 |  | 0.018 | 0.017 | 0.026 | 0.168 |
|  | 37034787 | cg23658326 |  | 0.007 | 0.007 | 0.010 | 0.353 |
|  | 37034814 | cg11600697 |  | 0.060 | 0.060 | 0.080 | 0.076 |
|  | 37034825 | cg21490561 |  | 0.036 | 0.036 | 0.037 | 0.997 |
|  | 37034840 | cg00893636 |  | 0.060 | 0.059 | 0.064 | 0.434 |
|  | 37034909 | cg03192963 |  | 0.050 | 0.050 | 0.054 | 0.586 |
|  | 37034956 | cg06791151 |  | 0.014 | 0.014 | 0.016 | 0.847 |
|  | 37034997 | cg07064226 |  | 0.050 | 0.052 | 0.063 | 0.448 |
|  | 37035063 | cg06108510 |  | 0.026 | 0.025 | 0.023 | 0.957 |
|  | 37035090 | cg24985459 |  | 0.001 | 0.004 | 0.001 | 0.090 |
|  | 37035117 | cg12790037 |  | 0.066 | 0.066 | 0.073 | 0.436 |
|  | 37035158 | cg25202636 |  | 0.046 | 0.052 | 0.053 | 0.225 |
|  | 37035168 | cg17621259 |  | 0.007 | 0.006 | 0.014 | 0.001 |
|  | 37035200 | cg14671526 |  | 0.007 | 0.007 | 0.013 | 0.391 |
|  | 37035205 | cg05906740 |  | 0.007 | 0.006 | 0.007 | 0.607 |
|  | 37035207 | cg27331401 |  | 0.059 | 0.059 | 0.080 | 0.018 |
|  | 37035220 | cg25837710 |  | 0.001 | 0.001 | 0.008 | 7.48E-06 |
|  | 37035222 | cg12851504 |  | 0.027 | 0.027 | 0.036 | 0.199 |
|  | 37035228 | cg06590608 |  | 0.006 | 0.005 | 0.016 | 4.54E-04 |
|  | 37035282 | cg11224603 |  | 0.012 | 0.011 | 0.018 | 0.238 |
|  | 37035345 | cg19208331 |  | 0.037 | 0.036 | 0.037 | 0.780 |
|  | 37035355 | cg14598950 |  | 0.023 | 0.023 | 0.025 | 0.926 |
|  | 37035399 | cg13846866 |  | 0.038 | 0.037 | 0.039 | 0.941 |
|  | 37036726 | cg04777024 |  | 0.885 | 0.890 | 0.872 | 0.093 |
|  | 37038591 | cg17024523 |  | 0.916 | 0.917 | 0.920 | 0.841 |
|  | 37048044 | ch.3.753362R |  | 0.149 | 0.142 | 0.153 | 0.191 |
|  | 37055414 | cg25212762 |  | 0.951 | 0.951 | 0.960 | 0.848 |
|  | 37082315 | cg11363877 |  | 0.940 | 0.939 | 0.942 | 0.771 |
|  | 37082380 | cg03405026 |  | 0.927 | 0.927 | 0.929 | 0.988 |
|  | 37092193 | cg16863190 |  | 0.912 | 0.902 | 0.925 | 0.741 |
|  | 37095036 | cg27373390 |  | 0.929 | 0.930 | 0.909 | 0.242 |
|  | 37152029 | cg01934787 |  | 0.918 | 0.912 | 0.913 | 0.695 |
|  | 37173546 | cg06284479 |  | 0.919 | 0.922 | 0.920 | 0.525 |
|  | 37179823 | cg24305555 |  | 0.939 | 0.944 | 0.939 | 0.248 |
|  | 37204814 | cg05433805 |  | 0.513 | 0.519 | 0.488 | 0.780 |
|  | 37212084 | cg15934958 |  | 0.884 | 0.870 | 0.884 | 0.174 |
|  | 37216510 | cg06734169 |  | 0.062 | 0.055 | 0.071 | 0.338 |
|  | 37217087 | cg12792366 |  | 0.042 | 0.045 | 0.026 | 0.356 |
|  | 37217675 | cg00747698 |  | 0.087 | 0.085 | 0.090 | 0.322 |
|  | 37217993 | cg22221026 |  | 0.005 | 0.005 | 0.007 | 0.703 |
|  | 37217996 | cg11574180 |  | 0.035 | 0.035 | 0.044 | 0.155 |
|  | 37218128 | cg09310383 |  | 0.110 | 0.109 | 0.114 | 0.614 |
|  | 37218150 | cg15011249 |  | 0.068 | 0.068 | 0.060 | 0.436 |
|  | 37218212 | cg17479303 |  | 0.040 | 0.037 | 0.032 | 0.199 |
|  | 37218771 | cg06853609 |  | 0.061 | 0.060 | 0.035 | 0.347 |
|  | 37219077 | cg22985146 |  | 0.491 | 0.506 | 0.506 | 0.268 |
|  | 37225266 | cg12999063 |  | 0.946 | 0.941 | 0.950 | 0.609 |
|  | 37239890 | cg11321190 |  | 0.612 | 0.619 | 0.609 | 0.865 |

Mean β value comparison of each genotype by ANOVA for the SNPs rs1800734, rs749072, and rs13098279 in CRC cases at 70 CpG sites in 3p21-3p22.
