# Supplementary material for: MLH1 Region Polymorphisms Show a Significant Association with CpG Island Shore Methylation in a Large Cohort of Healthy Individuals
Source: PLoS One. 2012 Dec 11;7(12):e51531. doi: 10.1371/journal.pone.0051531 (PMC3519863; doi:10.1371/journal.pone.0051531)
Supplement: Table S3 — Correlation between age and methylation. (DOCX) [file pone.0051531.s003.docx]

**Table S3.** Correlation between age and methylation.

| Shore Site Locations | Chromosome 3 Coordinate | Probe ID | CRC Cases (n=252) | R | P-value | Controls (n=846) | R | P-value |
| --- | --- | --- | --- | --- | --- | --- | --- | --- |
|  | 37018029 | cg21595053 |  | 0.089 | 0.186 |  | 0.025 | 0.482 |
| S1 | 37033373 | cg02103401 |  | 0.042 | 0.536 |  | -0.081 | 0.021 |
| S2 | 37033625 | cg24607398 |  | -0.037 | 0.582 |  | -0.085 | 0.016 |
| S3 | 37033632 | cg10990993 |  | -0.120 | 0.074 |  | -0.087 | 0.014 |
| S4 | 37033791 | cg04726821 |  | -0.209 | 0.002 |  | -0.170 | 1.30E-06 |
| S5 | 37033894 | cg11291081 |  | -0.115 | 0.089 |  | -0.101 | 0.004 |
| S6 | 37033903 | cg05670953 |  | -0.236 | 0.000 |  | -0.200 | 1.16E-08 |
| S7 | 37033980 | cg18320188 |  | 0.017 | 0.797 |  | -0.007 | 0.846 |
|  | 37034028 | cg04841293 |  | -0.139 | 0.039 |  | -0.036 | 0.302 |
|  | 37034066 | cg05845319 |  | -0.164 | 0.015 |  | -0.059 | 0.093 |
|  | 37034084 | cg21109167 |  | -0.258 | 0.000 |  | -0.181 | 2.58E-07 |
|  | 37034142 | cg03901257 |  | -0.171 | 0.011 |  | -0.066 | 0.062 |
|  | 37034154 | cg02279071 |  | -0.100 | 0.138 |  | -0.015 | 0.677 |
|  | 37034166 | cg14751544 |  | -0.073 | 0.278 |  | -0.006 | 0.864 |
|  | 37034346 | cg16764580 |  | 0.076 | 0.263 |  | 0.029 | 0.418 |
|  | 37034441 | cg01302270 |  | -0.017 | 0.805 |  | 0.039 | 0.274 |
|  | 37034473 | cg17641046 |  | 0.110 | 0.103 |  | 0.072 | 0.041 |
|  | 37034495 | cg07101782 |  | 0.044 | 0.516 |  | 0.057 | 0.105 |
|  | 37034654 | cg03497419 |  | -0.130 | 0.053 |  | 0.022 | 0.533 |
|  | 37034661 | cg27586588 |  | -0.099 | 0.141 |  | -0.002 | 0.966 |
|  | 37034693 | cg16433211 |  | 0.064 | 0.346 |  | 0.034 | 0.333 |
|  | 37034730 | cg10769891 |  | 0.005 | 0.940 |  | 0.056 | 0.111 |
|  | 37034739 | cg19132762 |  | -0.007 | 0.919 |  | -0.022 | 0.540 |
|  | 37034787 | cg23658326 |  | 0.063 | 0.349 |  | 0.017 | 0.625 |
|  | 37034814 | cg11600697 |  | 0.100 | 0.139 |  | 0.069 | 0.050 |
|  | 37034825 | cg21490561 |  | -0.133 | 0.048 |  | -0.011 | 0.759 |
|  | 37034840 | cg00893636 |  | -0.016 | 0.808 |  | -0.006 | 0.876 |
|  | 37034909 | cg03192963 |  | -0.059 | 0.380 |  | 0.021 | 0.546 |
|  | 37034956 | cg06791151 |  | -0.056 | 0.405 |  | -0.009 | 0.798 |
|  | 37034997 | cg07064226 |  | -0.127 | 0.059 |  | 0.000 | 0.991 |
|  | 37035063 | cg06108510 |  | -0.052 | 0.441 |  | 0.009 | 0.809 |
|  | 37035090 | cg24985459 |  | -0.046 | 0.497 |  | -0.017 | 0.635 |
|  | 37035117 | cg12790037 |  | -0.070 | 0.299 |  | 0.000 | 0.992 |
|  | 37035158 | cg25202636 |  | 0.065 | 0.336 |  | -0.002 | 0.955 |
|  | 37035168 | cg17621259 |  | 0.048 | 0.478 |  | 0.001 | 0.980 |
|  | 37035200 | cg14671526 |  | 0.044 | 0.513 |  | -0.001 | 0.974 |
|  | 37035205 | cg05906740 |  | 0.073 | 0.280 |  | -0.026 | 0.455 |
|  | 37035207 | cg27331401 |  | 0.061 | 0.363 |  | 0.051 | 0.152 |
|  | 37035220 | cg25837710 |  | -0.117 | 0.082 |  | 0.005 | 0.879 |
|  | 37035222 | cg12851504 |  | 0.000 | 0.997 |  | 0.063 | 0.076 |
|  | 37035228 | cg06590608 |  | 0.009 | 0.895 |  | -0.007 | 0.842 |
|  | 37035282 | cg11224603 |  | -0.044 | 0.512 |  | -0.028 | 0.430 |
|  | 37035345 | cg19208331 |  | -0.001 | 0.984 |  | 0.069 | 0.052 |
|  | 37035355 | cg14598950 |  | -0.097 | 0.151 |  | 0.015 | 0.672 |
|  | 37035399 | cg13846866 |  | -0.142 | 0.035 |  | 0.001 | 0.987 |
|  | 37036726 | cg04777024 |  | 0.060 | 0.373 |  | -0.034 | 0.342 |
|  | 37038591 | cg17024523 |  | 0.001 | 0.983 |  | 0.008 | 0.828 |
|  | 37048044 | ch.3.753362R |  | -0.121 | 0.073 |  | 0.067 | 0.056 |
|  | 37055414 | cg25212762 |  | -0.013 | 0.843 |  | 0.000 | 0.998 |
|  | 37082315 | cg11363877 |  | -0.041 | 0.540 |  | 0.001 | 0.972 |
|  | 37082380 | cg03405026 |  | 0.002 | 0.980 |  | -0.049 | 0.164 |
|  | 37092193 | cg16863190 |  | 0.058 | 0.395 |  | 0.032 | 0.368 |
|  | 37095036 | cg27373390 |  | 0.063 | 0.354 |  | -0.012 | 0.730 |
|  | 37152029 | cg01934787 |  | 0.031 | 0.649 |  | 0.025 | 0.485 |
|  | 37173546 | cg06284479 |  | -0.096 | 0.153 |  | -0.084 | 0.018 |
|  | 37179823 | cg24305555 |  | -0.003 | 0.966 |  | -0.011 | 0.752 |
|  | 37204814 | cg05433805 |  | -0.215 | 0.001 |  | -0.123 | 0.001 |
|  | 37212084 | cg15934958 |  | -0.252 | 0.000 |  | -0.156 | 9.15E-06 |
|  | 37216510 | cg06734169 |  | -0.160 | 0.017 |  | -0.131 | 1.89E-04 |
|  | 37217087 | cg12792366 |  | -0.118 | 0.080 |  | -0.055 | 0.117 |
|  | 37217675 | cg00747698 |  | -0.002 | 0.971 |  | 0.042 | 0.238 |
|  | 37217993 | cg22221026 |  | -0.066 | 0.330 |  | 0.050 | 0.156 |
|  | 37217996 | cg11574180 |  | 0.064 | 0.346 |  | 0.047 | 0.186 |
|  | 37218128 | cg09310383 |  | 0.017 | 0.799 |  | 0.021 | 0.546 |
|  | 37218150 | cg15011249 |  | -0.100 | 0.139 |  | -0.004 | 0.904 |
|  | 37218212 | cg17479303 |  | 0.052 | 0.442 |  | -0.007 | 0.850 |
|  | 37218771 | cg06853609 |  | -0.099 | 0.140 |  | -0.038 | 0.278 |
|  | 37219077 | cg22985146 |  | -0.232 | 0.001 |  | -0.142 | 5.43E-05 |
|  | 37225266 | cg12999063 |  | -0.075 | 0.270 |  | 0.003 | 0.924 |
|  | 37239890 | cg11321190 |  | -0.262 | 0.000 |  | -0.130 | 2.31E-04 |

Pearson correlation (R) and p-value between methylation and age at study recruitment, controlling for sex, for CRC cases and controls at 70 CpG sites in 3p21-3p22.
