# Supplementary material for: MLH1 Region Polymorphisms Show a Significant Association with CpG Island Shore Methylation in a Large Cohort of Healthy Individuals
Source: PLoS One. 2012 Dec 11;7(12):e51531. doi: 10.1371/journal.pone.0051531 (PMC3519863; doi:10.1371/journal.pone.0051531)
Supplement: Table S4 — Logistic regression analysis for gender. (DOCX) [file pone.0051531.s004.docx]

**Table S4.** Logistic regression analysis for gender.

| Shore Site Locations | Chromosome 3 Coordinate | Probe ID | Female Mean Methylation (n=476) | Male Mean Methylation (n=617) | P-value | Effect Size | Lower 95% CI | Upper 95% CI |
| --- | --- | --- | --- | --- | --- | --- | --- | --- |
|  | 37018029 | cg21595053 | 0.933 | 0.933 | 0.911 | 0.995 | 0.910 | 1.088 |
| S1 | 37033373 | cg02103401 | 0.632 | 0.632 | 0.058 | 1.014 | 1.000 | 1.028 |
| S2 | 37033625 | cg24607398 | 0.775 | 0.775 | 0.433 | 1.008 | 0.988 | 1.029 |
| S3 | 37033632 | cg10990993 | 0.745 | 0.745 | 0.624 | 1.006 | 0.984 | 1.028 |
| S4 | 37033791 | cg04726821 | 0.244 | 0.244 | 0.680 | 1.005 | 0.982 | 1.029 |
| S5 | 37033894 | cg11291081 | 0.118 | 0.118 | 0.001 | 0.939 | 0.905 | 0.974 |
| S6 | 37033903 | cg05670953 | 0.195 | 0.195 | 0.003 | 0.939 | 0.917 | 0.962 |
| S7 | 37033980 | cg18320188 | 0.120 | 0.120 | 0.030 | 0.938 | 0.885 | 0.994 |
|  | 37034028 | cg04841293 | 0.051 | 0.051 | 0.056 | 0.888 | 0.785 | 1.003 |
|  | 37034066 | cg05845319 | 0.074 | 0.074 | 0.002 | 0.838 | 0.778 | 0.902 |
|  | 37034084 | cg21109167 | 0.167 | 0.167 | 0.000 | 0.911 | 0.881 | 0.942 |
|  | 37034142 | cg03901257 | 0.045 | 0.045 | 0.086 | 0.917 | 0.832 | 1.012 |
|  | 37034154 | cg02279071 | 0.035 | 0.035 | 0.186 | 0.934 | 0.845 | 1.033 |
|  | 37034166 | cg14751544 | 0.063 | 0.063 | 0.531 | 0.974 | 0.898 | 1.057 |
|  | 37034346 | cg16764580 | 0.023 | 0.023 | 0.667 | 0.989 | 0.938 | 1.042 |
|  | 37034441 | cg01302270 | 0.061 | 0.061 | 0.699 | 1.019 | 0.926 | 1.121 |
|  | 37034473 | cg17641046 | 0.065 | 0.065 | 0.020 | 1.081 | 1.012 | 1.154 |
|  | 37034495 | cg07101782 | 0.004 | 0.004 | 0.200 | 1.225 | 0.898 | 1.670 |
|  | 37034654 | cg03497419 | 0.027 | 0.027 | 0.752 | 0.986 | 0.901 | 1.078 |
|  | 37034661 | cg27586588 | 0.035 | 0.035 | 0.325 | 1.051 | 0.952 | 1.161 |
|  | 37034693 | cg16433211 | 0.014 | 0.014 | 0.086 | 1.128 | 0.983 | 1.295 |
|  | 37034730 | cg10769891 | 0.025 | 0.025 | 0.517 | 1.051 | 0.905 | 1.219 |
|  | 37034739 | cg19132762 | 0.019 | 0.019 | 0.008 | 1.152 | 1.038 | 1.279 |
|  | 37034787 | cg23658326 | 0.007 | 0.007 | 0.764 | 1.027 | 0.863 | 1.222 |
|  | 37034814 | cg11600697 | 0.061 | 0.061 | 0.282 | 1.043 | 0.966 | 1.126 |
|  | 37034825 | cg21490561 | 0.039 | 0.039 | 0.141 | 1.079 | 0.975 | 1.193 |
|  | 37034840 | cg00893636 | 0.059 | 0.059 | 0.017 | 0.843 | 0.732 | 0.970 |
|  | 37034909 | cg03192963 | 0.050 | 0.050 | 0.823 | 0.985 | 0.863 | 1.124 |
|  | 37034956 | cg06791151 | 0.015 | 0.015 | 0.059 | 1.265 | 0.991 | 1.615 |
|  | 37034997 | cg07064226 | 0.051 | 0.051 | 0.221 | 0.966 | 0.913 | 1.021 |
|  | 37035063 | cg06108510 | 0.025 | 0.025 | 0.600 | 1.021 | 0.945 | 1.103 |
|  | 37035090 | cg24985459 | 0.002 | 0.002 | 0.750 | 0.971 | 0.812 | 1.162 |
|  | 37035117 | cg12790037 | 0.067 | 0.067 | 0.627 | 1.029 | 0.916 | 1.156 |
|  | 37035158 | cg25202636 | 0.049 | 0.049 | 0.470 | 1.020 | 0.966 | 1.077 |
|  | 37035168 | cg17621259 | 0.006 | 0.006 | 0.796 | 1.031 | 0.816 | 1.305 |
|  | 37035200 | cg14671526 | 0.006 | 0.006 | 0.324 | 0.918 | 0.773 | 1.089 |
|  | 37035205 | cg05906740 | 0.006 | 0.006 | 0.425 | 1.082 | 0.891 | 1.315 |
|  | 37035207 | cg27331401 | 0.060 | 0.060 | 0.071 | 1.085 | 0.993 | 1.186 |
|  | 37035220 | cg25837710 | 0.001 | 0.001 | 0.486 | 0.874 | 0.599 | 1.276 |
|  | 37035222 | cg12851504 | 0.028 | 0.028 | 0.004 | 1.201 | 1.059 | 1.361 |
|  | 37035228 | cg06590608 | 0.006 | 0.006 | 0.519 | 1.074 | 0.865 | 1.334 |
|  | 37035282 | cg11224603 | 0.011 | 0.011 | 0.930 | 1.008 | 0.851 | 1.192 |
|  | 37035345 | cg19208331 | 0.038 | 0.038 | 0.083 | 1.102 | 0.988 | 1.229 |
|  | 37035355 | cg14598950 | 0.024 | 0.024 | 0.295 | 0.950 | 0.863 | 1.046 |
|  | 37035399 | cg13846866 | 0.037 | 0.037 | 0.525 | 0.986 | 0.943 | 1.030 |
|  | 37036726 | cg04777024 | 0.885 | 0.885 | 0.780 | 0.992 | 0.937 | 1.050 |
|  | 37038591 | cg17024523 | 0.912 | 0.912 | 0.063 | 0.942 | 0.884 | 1.003 |
|  | 37048044 | ch.3.753362R | 0.145 | 0.145 | 0.092 | 0.962 | 0.919 | 1.006 |
|  | 37055414 | cg25212762 | 0.953 | 0.953 | 0.256 | 1.028 | 0.980 | 1.077 |
|  | 37082315 | cg11363877 | 0.938 | 0.938 | 0.218 | 0.956 | 0.890 | 1.027 |
|  | 37082380 | cg03405026 | 0.928 | 0.928 | 0.308 | 1.044 | 0.961 | 1.134 |
|  | 37092193 | cg16863190 | 0.925 | 0.925 | 0.017 | 1.022 | 1.004 | 1.041 |
|  | 37095036 | cg27373390 | 0.927 | 0.927 | 0.333 | 1.023 | 0.976 | 1.073 |
|  | 37152029 | cg01934787 | 0.918 | 0.918 | 0.469 | 0.989 | 0.960 | 1.019 |
|  | 37173546 | cg06284479 | 0.919 | 0.919 | 0.742 | 0.990 | 0.935 | 1.049 |
|  | 37179823 | cg24305555 | 0.940 | 0.940 | 0.588 | 1.015 | 0.962 | 1.070 |
|  | 37204814 | cg05433805 | 0.512 | 0.512 | 0.058 | 0.988 | 0.976 | 1.000 |
|  | 37212084 | cg15934958 | 0.877 | 0.877 | 0.352 | 0.989 | 0.966 | 1.012 |
|  | 37216510 | cg06734169 | 0.059 | 0.059 | 0.103 | 0.962 | 0.918 | 1.008 |
|  | 37217087 | cg12792366 | 0.044 | 0.044 | 0.801 | 1.007 | 0.956 | 1.060 |
|  | 37217675 | cg00747698 | 0.086 | 0.086 | 0.904 | 0.994 | 0.900 | 1.097 |
|  | 37217993 | cg22221026 | 0.005 | 0.005 | 0.242 | 0.838 | 0.623 | 1.127 |
|  | 37217996 | cg11574180 | 0.036 | 0.036 | 0.298 | 1.072 | 0.941 | 1.221 |
|  | 37218128 | cg09310383 | 0.108 | 0.108 | 0.234 | 0.944 | 0.858 | 1.038 |
|  | 37218150 | cg15011249 | 0.069 | 0.069 | 0.486 | 1.036 | 0.938 | 1.144 |
|  | 37218212 | cg17479303 | 0.039 | 0.039 | 0.270 | 0.960 | 0.892 | 1.032 |
|  | 37218771 | cg06853609 | 0.060 | 0.060 | 0.064 | 0.967 | 0.933 | 1.002 |
|  | 37219077 | cg22985146 | 0.491 | 0.491 | 0.180 | 0.988 | 0.971 | 1.006 |
|  | 37225266 | cg12999063 | 0.942 | 0.942 | 0.295 | 1.013 | 0.989 | 1.037 |
|  | 37239890 | cg11321190 | 0.620 | 0.620 | 0.042 | 0.984 | 0.969 | 0.999 |

Mean β value of is shown for males and females along with logistic regression analysis at seven CpG sites in the *MLH1* CpG island shore. Analysis of male versus female methylation is adjusted for age.
