# Supplementary material for: MLH1 Region Polymorphisms Show a Significant Association with CpG Island Shore Methylation in a Large Cohort of Healthy Individuals
Source: PLoS One. 2012 Dec 11;7(12):e51531. doi: 10.1371/journal.pone.0051531 (PMC3519863; doi:10.1371/journal.pone.0051531)
Supplement: Table S5 — Regression analysis for CRC cases vs. controls. (DOCX) [file pone.0051531.s005.docx]

**Table S5**. Regression analysis for CRC cases vs. controls.

| Shore Site Locations | Chromosome 3 Coordinate | Probe ID | Control Mean Methylation (n=846) | CRC Case Mean Methylation (n=252) | P-value | Effect Size | Lower 95% CI | Upper 95% CI |
| --- | --- | --- | --- | --- | --- | --- | --- | --- |
|  | 37018029 | cg21595053 | 0.932 | 0.935 | 0.010 | 0.870 | 0.783 | 0.967 |
| S1 | 37033373 | cg02103401 | 0.630 | 0.620 | 0.109 | 1.014 | 0.997 | 1.031 |
| S2 | 37033625 | cg24607398 | 0.775 | 0.770 | 0.182 | 1.016 | 0.992 | 1.041 |
| S3 | 37033632 | cg10990993 | 0.747 | 0.738 | 0.017 | 1.032 | 1.006 | 1.059 |
| S4 | 37033791 | cg04726821 | 0.245 | 0.238 | 0.040 | 1.031 | 1.001 | 1.060 |
| S5 | 37033894 | cg11291081 | 0.122 | 0.121 | 0.464 | 1.016 | 0.973 | 1.061 |
| S6 | 37033903 | cg05670953 | 0.204 | 0.199 | 0.058 | 1.028 | 0.999 | 1.057 |
| S7 | 37033980 | cg18320188 | 0.121 | 0.121 | 0.768 | 1.010 | 0.943 | 1.082 |
|  | 37034028 | cg04841293 | 0.052 | 0.050 | 0.024 | 1.183 | 1.023 | 1.367 |
|  | 37034066 | cg05845319 | 0.077 | 0.076 | 0.263 | 1.049 | 0.965 | 1.141 |
|  | 37034084 | cg21109167 | 0.174 | 0.170 | 0.045 | 1.041 | 1.001 | 1.082 |
|  | 37034142 | cg03901257 | 0.046 | 0.044 | 0.005 | 1.179 | 1.050 | 1.323 |
|  | 37034154 | cg02279071 | 0.036 | 0.035 | 0.028 | 1.143 | 1.015 | 1.287 |
|  | 37034166 | cg14751544 | 0.064 | 0.062 | 0.026 | 1.114 | 1.013 | 1.225 |
|  | 37034346 | cg16764580 | 0.023 | 0.026 | 0.013 | 0.927 | 0.872 | 0.984 |
|  | 37034441 | cg01302270 | 0.061 | 0.061 | 0.825 | 0.987 | 0.882 | 1.105 |
|  | 37034473 | cg17641046 | 0.063 | 0.064 | 0.681 | 0.984 | 0.911 | 1.063 |
|  | 37034495 | cg07101782 | 0.004 | 0.004 | 0.840 | 0.968 | 0.706 | 1.327 |
|  | 37034654 | cg03497419 | 0.027 | 0.028 | 0.450 | 0.960 | 0.863 | 1.067 |
|  | 37034661 | cg27586588 | 0.034 | 0.034 | 0.354 | 1.057 | 0.940 | 1.189 |
|  | 37034693 | cg16433211 | 0.014 | 0.012 | 0.087 | 1.158 | 0.979 | 1.370 |
|  | 37034730 | cg10769891 | 0.025 | 0.025 | 0.679 | 1.038 | 0.869 | 1.241 |
|  | 37034739 | cg19132762 | 0.019 | 0.018 | 0.517 | 1.041 | 0.922 | 1.176 |
|  | 37034787 | cg23658326 | 0.007 | 0.007 | 0.269 | 0.900 | 0.747 | 1.084 |
|  | 37034814 | cg11600697 | 0.061 | 0.060 | 0.952 | 0.997 | 0.911 | 1.092 |
|  | 37034825 | cg21490561 | 0.039 | 0.037 | 0.010 | 1.170 | 1.038 | 1.318 |
|  | 37034840 | cg00893636 | 0.059 | 0.060 | 0.502 | 0.945 | 0.800 | 1.115 |
|  | 37034909 | cg03192963 | 0.050 | 0.050 | 0.589 | 0.958 | 0.820 | 1.119 |
|  | 37034956 | cg06791151 | 0.015 | 0.014 | 0.091 | 1.287 | 0.961 | 1.723 |
|  | 37034997 | cg07064226 | 0.051 | 0.051 | 0.668 | 1.015 | 0.949 | 1.084 |
|  | 37035063 | cg06108510 | 0.025 | 0.026 | 0.394 | 0.962 | 0.879 | 1.052 |
|  | 37035090 | cg24985459 | 0.002 | 0.002 | 0.238 | 0.891 | 0.736 | 1.079 |
|  | 37035117 | cg12790037 | 0.067 | 0.067 | 0.273 | 1.081 | 0.941 | 1.241 |
|  | 37035158 | cg25202636 | 0.048 | 0.049 | 0.683 | 0.987 | 0.926 | 1.052 |
|  | 37035168 | cg17621259 | 0.006 | 0.007 | 0.241 | 0.855 | 0.658 | 1.111 |
|  | 37035200 | cg14671526 | 0.006 | 0.007 | 0.013 | 0.785 | 0.648 | 0.950 |
|  | 37035205 | cg05906740 | 0.006 | 0.007 | 0.166 | 0.854 | 0.683 | 1.068 |
|  | 37035207 | cg27331401 | 0.059 | 0.060 | 0.591 | 0.973 | 0.879 | 1.076 |
|  | 37035220 | cg25837710 | 0.001 | 0.002 | 0.406 | 0.846 | 0.570 | 1.256 |
|  | 37035222 | cg12851504 | 0.027 | 0.027 | 0.596 | 1.040 | 0.899 | 1.204 |
|  | 37035228 | cg06590608 | 0.006 | 0.006 | 0.980 | 1.003 | 0.779 | 1.293 |
|  | 37035282 | cg11224603 | 0.011 | 0.012 | 0.027 | 0.803 | 0.662 | 0.975 |
|  | 37035345 | cg19208331 | 0.038 | 0.037 | 0.378 | 1.060 | 0.931 | 1.207 |
|  | 37035355 | cg14598950 | 0.025 | 0.023 | 0.147 | 1.116 | 0.962 | 1.295 |
|  | 37035399 | cg13846866 | 0.037 | 0.038 | 0.593 | 0.986 | 0.937 | 1.038 |
|  | 37036726 | cg04777024 | 0.885 | 0.886 | 0.246 | 0.961 | 0.898 | 1.028 |
|  | 37038591 | cg17024523 | 0.913 | 0.916 | 0.014 | 0.911 | 0.845 | 0.982 |
|  | 37048044 | ch.3.753362R | 0.146 | 0.147 | 0.462 | 0.980 | 0.929 | 1.034 |
|  | 37055414 | cg25212762 | 0.953 | 0.951 | 0.560 | 1.016 | 0.962 | 1.074 |
|  | 37082315 | cg11363877 | 0.938 | 0.940 | 0.255 | 0.952 | 0.875 | 1.036 |
|  | 37082380 | cg03405026 | 0.927 | 0.927 | 0.638 | 0.977 | 0.886 | 1.077 |
|  | 37092193 | cg16863190 | 0.923 | 0.910 | 0.016 | 1.023 | 1.004 | 1.043 |
|  | 37095036 | cg27373390 | 0.926 | 0.928 | 0.159 | 0.958 | 0.903 | 1.017 |
|  | 37152029 | cg01934787 | 0.919 | 0.917 | 0.395 | 1.015 | 0.981 | 1.050 |
|  | 37173546 | cg06284479 | 0.919 | 0.919 | 0.861 | 0.994 | 0.928 | 1.065 |
|  | 37179823 | cg24305555 | 0.940 | 0.941 | 0.466 | 0.977 | 0.917 | 1.040 |
|  | 37204814 | cg05433805 | 0.519 | 0.514 | 0.265 | 1.008 | 0.994 | 1.024 |
|  | 37212084 | cg15934958 | 0.879 | 0.879 | 0.844 | 1.003 | 0.975 | 1.031 |
|  | 37216510 | cg06734169 | 0.060 | 0.060 | 0.657 | 1.012 | 0.959 | 1.069 |
|  | 37217087 | cg12792366 | 0.044 | 0.043 | 0.261 | 1.037 | 0.973 | 1.104 |
|  | 37217675 | cg00747698 | 0.086 | 0.086 | 0.910 | 1.007 | 0.896 | 1.132 |
|  | 37217993 | cg22221026 | 0.005 | 0.005 | 0.876 | 1.028 | 0.724 | 1.462 |
|  | 37217996 | cg11574180 | 0.036 | 0.035 | 0.333 | 1.080 | 0.925 | 1.260 |
|  | 37218128 | cg09310383 | 0.109 | 0.110 | 0.114 | 0.914 | 0.818 | 1.022 |
|  | 37218150 | cg15011249 | 0.070 | 0.068 | 0.055 | 1.123 | 0.998 | 1.265 |
|  | 37218212 | cg17479303 | 0.039 | 0.039 | 0.453 | 1.034 | 0.948 | 1.126 |
|  | 37218771 | cg06853609 | 0.062 | 0.060 | 0.488 | 1.015 | 0.973 | 1.059 |
|  | 37219077 | cg22985146 | 0.493 | 0.497 | 0.441 | 0.992 | 0.971 | 1.013 |
|  | 37225266 | cg12999063 | 0.939 | 0.944 | 0.160 | 0.974 | 0.939 | 1.010 |
|  | 37239890 | cg11321190 | 0.628 | 0.614 | 0.004 | 1.028 | 1.009 | 1.047 |

Mean methylation and binomial logistic regression analysis for CRC cases and controls for 70 CpG sites in the MLH1 region, including p-value, effect size, and 95% confidence intervals. Adjusted for age and sex.
